# Andrographolide attenuates microglial senescence in Alzheimer’s disease mice by suppressing the STAT3 signaling

**DOI:** 10.1016/j.isci.2026.116033

**Published:** 2026-05-20

**Authors:** Haochang Song, Meng Yang, Shengquan Wu, Qihui Dai, Weihong Qin, Weiheng Xie, Yuzhi Chen, Xiaoyun Jiang, Xiaojun Zhang, Xiuqin Deng, Chuang Ouyang, Yunman Zhang, Xinguang Liu, Yingjie Zhu, Gonghua Huang

**Affiliations:** 1 Guangdong Provincial Key Laboratory of Medical Immunology and Molecular Diagnostics, The Affiliated Dongguan Songshan Lake Central Hospital, Guangdong Medical University, Dongguan 523808, China; 2 Affiliated Hospital of Guangdong Medical University, Zhanjiang 524001, China; 3 Shenzhen Institute of Advanced Technology, Chinese Academy of Sciences, Shenzhen 518055, China

**Keywords:** neuroscience, cell biology

## Abstract

Andrographolide (AP), a diterpenoid extracted from *Andrographis paniculata*, has emerged as a promising treatment for Alzheimer’s disease (AD) in preclinical studies, but the underlying mechanisms remain incompletely defined. Here, we demonstrated that AP treatment improved cognition performance and reduced amyloid-β (Aβ) plaque accumulation in 5×FAD transgenic mice of both sexes, by mitigating microglial senescence. Proteomic analysis revealed that AP markedly decreased cholesterol content in the cerebral cortex. Using an *in vitro* low-density lipoprotein-induced senescence model, we found that AP significantly alleviated senescence in BV2 microglia while enhancing their phagocytic capacity. Mechanistically, AP mitigated microglial senescence by inhibiting STAT3 signaling. Overall, these findings identify a previously unrecognized immunometabolic mechanism for AP in the treatment of AD.

## Highlights

• AP improves cognition function and reduces amyloid-β plaque burden in 5×FAD mice
• Cholesterol synthesis-related genes are significantly upregulated in 5×FAD mice
• AP attenuates cholesterol accumulation-induced microglial senescence

## Graphical abstract

**Figure:**
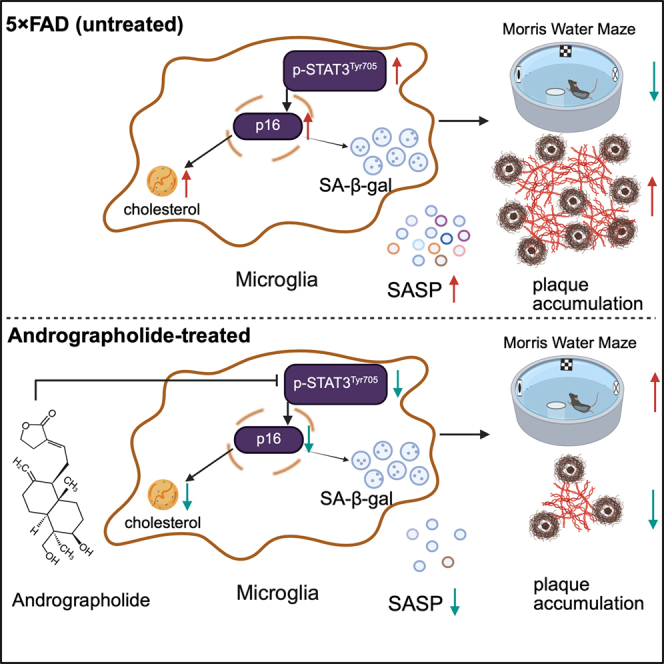

## Introduction

Alzheimer’s disease (AD), the most prevalent form of dementia among elderly individuals, is a progressive neurodegenerative disorder. It is featured by the presence of neurofibrillary tangles composed of hyperphosphorylated tau protein and the abnormal accumulation of amyloid β peptide (Aβ) in amyloid plaques.^1^ The incidence of AD has been rising along with the aging population.^2^ Accumulating evidence suggests that AD results from a complex interplay of genetic and environmental factors, leading to substantial challenges for the development of effective therapeutic strategies.^3^ Despite considerable research endeavors, recent anti-amyloid immunotherapies have demonstrated only modest clinical efficacy, including a delay in symptom progression; however, no effective pharmacological treatments for AD are currently available.^4^

Recent studies have demonstrated that cellular senescence in the brain significantly contributes to the pathogenesis of neurodegenerative diseases, particularly AD.^5^ Senescent cells are characterized by irreversible cell-cycle arrest and increased expression of cell cycle inhibitor markers, such as p16^Ink4a^ and p21^Cip1^, and the secretion of proinflammatory factors, collectively known as the senescence-associated secretory phenotype (SASP).^6^^,^^7^ The central nervous system (CNS) comprises various glial cell types, including microglia, astrocytes, and oligodendrocyte progenitor cells.^8^ Reducing senescent glial populations can significantly alleviate AD-related symptoms and pathology.^9^^,^^10^^,^^11^^,^^12^ Microglia, the resident macrophages of the CNS, actively sense extracellular changes and perform diverse regulatory roles.^13^^,^^14^ Under homeostatic conditions, microglia maintain neural health through synaptic pruning and the clearance of cellular debris, including protein aggregates and damaged myelin.^15^ However, recent studies suggest that senescent microglia may actively contribute to the progression of AD by promoting chronic inflammation and impairing clearance mechanisms.^16^^,^^17^ Thus, targeting microglial senescence represents a promising therapeutic strategy for AD.

Andrographolide (AP) is a diterpene lactone extracted from the medicinal plant *Andrographis paniculata*, which is known for its anti-inflammatory, antioxidant, anticancer, and immunomodulatory properties.^18^^,^^19^^,^^20^^,^^21^ As a small, apolar molecule, AP readily crosses the blood-brain barrier and has neuroprotective effects within the CNS.^22^^,^^23^^,^^24^ Our previous research demonstrated that AP effectively inhibits renal tubular cell senescence and suppresses the SASP. However, whether AP has similar effects in inhibiting microglial senescence remains unclear. In this study, both *in vivo* and *in vitro*, we have demonstrated that AP can significantly attenuate microglial senescence phenotypes, potentially by ameliorating cholesterol levels in a STAT3-dependent manner.

## Results

### AP treatment ameliorates cognitive impairment and reduces Aβ deposition in the cortex of 5×FAD mice

**Figure 1:**
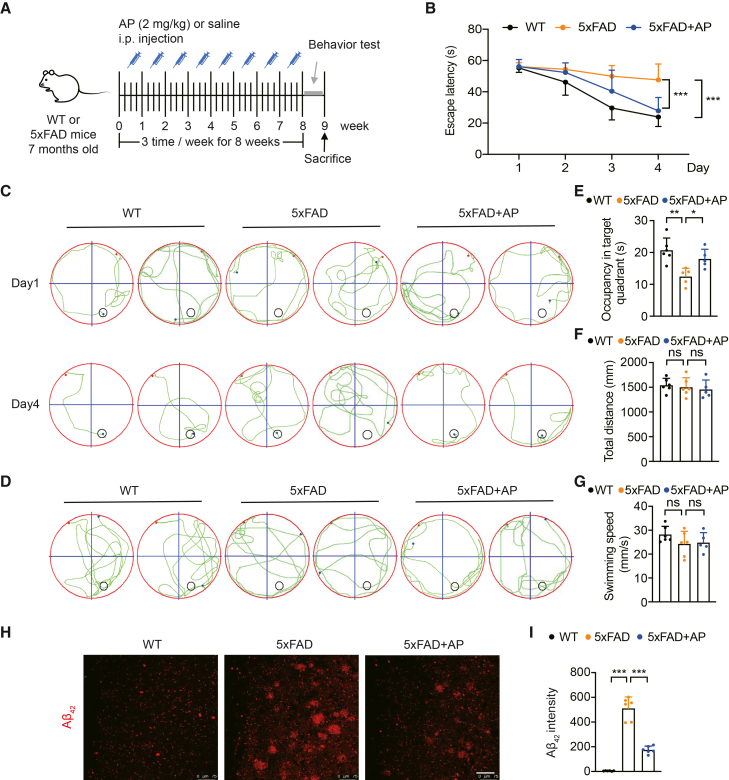
AP treatment ameliorates cognitive impairment and reduces Aβ deposition in the cortex of 5×FAD mice (A) Schematic of the experimental design for saline or AP injection into mice. ip: intraperitoneal. (B) Escape latency of mice treated or untreated with AP in the hidden platform task (*N* = 6). Each data point represents the test result of one mouse. (C) Representative movement paths of mice in the hidden platform task. (D) Representative movement paths of mice in the probe test. (E) Target quadrant time in the probe trial (*N* = 6). Each data point represents the test result of one mouse. (F) Swimming distance in the probe trial (*N* = 6). Each data point represents the test result of one mouse. (G) Swimming speed in the probe trial (*N* = 5). Each data point represents the test result of one mouse. (H) Immunofluorescence staining of anti-Aβ_42_ antibodies in the cortex. Scale bar, 75 μm. (I) Quantitative immunofluorescence staining of Aβ_42_ (*N* = 3, *n* = 2). Each data point represents the test result of one tissue slice. Data are expressed as mean ± SEM; N = the number of mice, n = number of fields of view per section from a single mouse. ANOVA followed by Bonferroni post hoc test was used. Statistical significance is indicated as ns: not significant, ∗*p* < 0.05, ∗∗*p* < 0.01, ∗∗∗*p* < 0.001.

To assess whether AP can alleviate cognitive impairments in 7-month-old 5×FAD mice, WT and 5×FAD animals were randomly allocated to AP treatment (2.0 mg/kg, i.p., three times weekly for 8 weeks) or saline vehicle control groups. Behavioral testing in the Morris water maze (MWM) was conducted with 5–6 mice per group (Figure 1A). The results showed that the escape latency was significantly longer in 5×FAD mice than that in WT controls. AP treatment significantly improved the escape latency of 5×FAD mice (Figures 1B and 1C), indicating that AP improved cognitive performance in 5×FAD mice. Although statistically significant differences were detected, we acknowledge that these relatively modest sample sizes may somewhat constrain the statistical power of the behavioral assessments. On day 6 of the probe test, 5×FAD mice spent less time in the target quadrant than WT mice.

In contrast, AP-treated 5×FAD mice presented a significantly increased time in the target quadrant (Figures 1D and 1E), indicating that AP can also improve memory in 5×FAD mice. Notably, no significant differences were observed in swimming distance or swimming speed among the 3 groups of experimental mice (Figures 1F and 1G). The abnormal aggregation of Aβ peptides and the subsequent formation of senile plaques pathologically characterizes AD.^24^ To assess the impact of AP on AD-like pathology, immunofluorescence staining was performed on cortical sections to detect Aβ_42_ expression. The results showed that 5×FAD mice presented prominent Aβ_42_ deposition, whereas WT mice presented no detectable Aβ_42_ accumulation, and AP treatment markedly reduced the Aβ_42_ plaque-positive area and overall plaque density in the cortex of 5×FAD mice (Figures 1H and 1I). Moreover, we measured the levels of Aβ_40_ and Aβ_42_ in the cortex by ELISA and found that both Aβ_40_ and Aβ_42_ were significantly lower in AP-treated 5×FAD mice than in 5×FAD mice (Figure S1A).

Additionally, we observed a positive effect of AP in preventing the toxic Aβ production, as indicated by the decreasing expression of amyloid precursor protein (APP) and β-secretase (BACE1) in 5×FAD mice (Figures S1B and S1C). Given that toxic Aβ was mainly generated by BACE1-mediated APP cleavage, we reasoned that AP’s neuroprotective effect in reducing Aβ could be achieved by decreasing APP expression or inhibiting BACE-mediated cleavage. Together, these findings suggest that AP can improve cognitive function and diminish plaque accumulation in the cortical region in 5×FAD mice.

### AP treatment mitigates microglial senescence in 5×FAD mice

**Figure 2:**
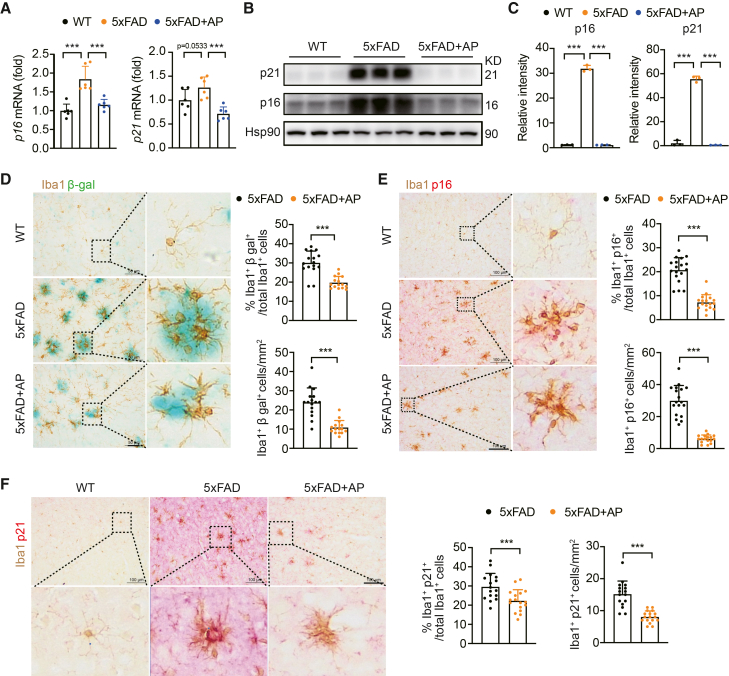
AP inhibits cellular senescence of microglia (A) Quantitative PCR analysis of p16 and p21 in the cortex tissue (*N* = 6). Each data point represents the test result of one mouse. (B) Western blot analysis of p16 and p21 expression in the cortex tissue (*N* = 3). Each data lane represents the test result of one mouse. (C) Quantitative data of p16 and p21 protein levels (*N* = 3). Each data point represents the test result of one mouse. (D) Immunohistochemistry staining with anti-Iba1 antibody and β-gal staining, as well as quantitative statistics, in the cortex. Scale bar, 50 μm (5×FAD: *N* = 5, n = 3–4; 5×FAD+AP: *N* = 5, *n* = 3). Each data point represents the test result of one tissue slice. Sections were collected at approximately Bregma −1.70 mm (or immediately adjacent levels). (E) Immunohistochemistry staining with anti-Iba1 and anti-p16 antibodies, as well as quantitative statistics, in the cortex. Scale bar = 100 μm (5×FAD: *N* = 5, n = 3–4; 5×FAD+AP: *N* = 5, n = 3–4). Each data point represents the test result of one tissue slice. Sections were collected at approximately Bregma −1.70 mm (or immediately adjacent levels). (F) Immunohistochemistry staining with anti-Iba1 and anti-p21 antibodies, as well as quantitative statistics, in the cortex. Scale bar, 100 μm (5×FAD: *N* = 5, n = 3–4; 5×FAD+AP: *N* = 5, n = 3–4). Sections were collected at approximately Bregma −1.70 mm (or immediately adjacent levels). Each data point represents the test result of one tissue slice. Data are expressed as mean ± SEM; *N* = the number of mice, *n* = number of fields of view per section from a single mouse. ANOVA followed by Bonferroni post hoc test, and unpaired Student’s *t* test were used. Statistical significance is indicated as ∗∗∗*p* < 0.001.

Given the established role of cellular senescence in CNS tissue dysfunction, we investigated the impact of AP on this process in 5×FAD mice. RT-qPCR and Western blot analyses revealed significantly upregulated expression of the cyclin-dependent kinase inhibitors p16 and p21 in the 5×FAD group compared with the control group (Figures 2A‒2C); however, AP treatment significantly reduced the expression of p16 and p21 at both mRNA and protein levels in 5×FAD mice (Figures 2A‒2C). Co-localization analysis of SA-β-gal with the microglial marker Iba1 revealed that microglia were the predominant senescent cells in the cerebral cortex of 5×FAD mice. This co-localization was significantly decreased after AP treatment (Figure 2D). Consistent with these findings, immunohistochemistry analysis revealed an increased co-localization of p16 and p21 with Iba1-positive cells in 5×FAD mice, and this effect was also significantly reduced after AP administration (Figures 2E, 2F, S1D, and S1E). Notably, we observed a significant increase in Iba1^+^CD68^+^ microglia, as well as in the ratio of Aβ plaque engulfed by CD68 to total CD68, following treatment with AP (Figures S1F and S1G), suggesting an enhanced microglial phagocytic activity. Moreover, our findings showed a notable co-localization of activated microglia and senescent staining within the dentate gyrus of 5×FAD mice. In contrast, senescent staining was less pronounced in the CA1 region; however, in the CA3 region, there was a distinct co-localization of activated microglia and senescent staining (Figure S1H). Together, these findings demonstrate that AP effectively mitigates cellular senescence, particularly in microglia, potentially contributing to the improved microglial function.

The disease-associated microglia (DAM) are involved in AD pathology.^25^ We next examined the effect of AP on this specific microglial subpopulation in 5×FAD mice. Co-localization analysis of SA-β-gal with the DAM marker Dectin-1 revealed a clear association of senescence with DAM in 5×FAD mice, a phenomenon largely absent in WT controls (Figure S2A). Importantly, AP administration significantly decreased this co-localization (Figure S2A), which was further supported by immunohistochemical staining showing reduced co-localization of Dectin-1 with p16 and p21 following AP treatment (Figures S2B and S2C). Thus, AP treatment effectively reduces the senescence-associated phenotype of DAM in the 5×FAD mouse brain.

### AP treatment reduces the activation of astrocytes and microglia in 5×FAD mice

**Figure 3:**
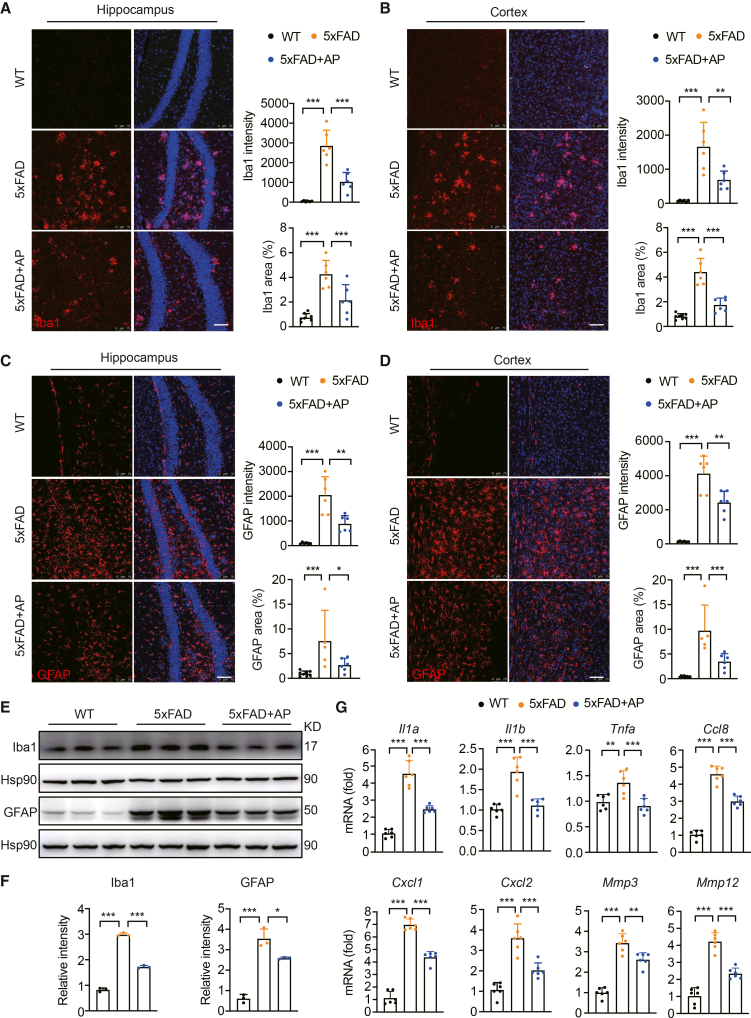
AP reduces the activation of microglia and astrocytes (A) Immunofluorescence staining with anti-Iba1 antibody and quantification in the hippocampus. Scale bar, 75 μm (*N* = 3, *n* = 2). Each data point represents the test result of one tissue slice. (B) Immunofluorescence staining with anti-Iba1 antibody and quantification in the cortex. Scale bar, 75 μm (*N* = 3, *n* = 2). Each data point represents the test result of one tissue slice. (C) Immunofluorescence staining with anti-GFAP antibody and quantification in the hippocampus. Scale bar, 75 μm (*N* = 3, *n* = 2). Each data point represents the test result of one tissue slice. (D) Immunofluorescence staining with anti-GFAP antibody and quantification in the cortex. Scale bar = 75 μm (*N* = 3, *n* = 2). Each data point represents the test result of one tissue slice. (E) Western blot analysis of Iba1 and GFAP expression in the cortex tissue (*N* = 3). Each data lane represents the test result of one mouse. (F) Quantitative data of Iba1 and GFAP protein levels (*N* = 3). Each data point represents the test result of one mouse. (G) Quantitative PCR analysis of SASP factors in the cortex tissue (*N* = 3). Each data point represents the test result of one mouse. Data are expressed as mean ± SEM; *N* = the number of mice, *n* = number of fields of view per section from a single mouse. ANOVA followed by Bonferroni post hoc test was used. Statistical significance is indicated as ∗*p* < 0.05, ∗∗*p* < 0.01, ∗∗∗*p* < 0.001.

Neuroinflammation is considered a pivotal pathological hallmark of AD, and microglia have been shown to play pivotal roles in initiating defense mechanisms against neuroinflammation.^26^ Although the number of microglial branches in AP-treated 5×FAD mice showed no significant increase, the relative area of the cell bodies was markedly reduced compared to that in untreated 5×FAD mice (Figure S2D). We observed a high presence of microglia in both the hippocampus and cortex in 5×FAD mice, whereas these Iba1-positive microglia were rarely observed in WT mice (Figures 3A and 3B). Treatment with AP led to a significant reduction in Iba1 immunofluorescence intensity in both the cortex and the hippocampus in 5×FAD mice (Figures 3A and 3B). Astrocytes, which are known to be primarily for their diverse support functions within the CNS, are activated in response to inflammatory stimuli and microglial interactions.^27^ We quantified astrocyte activation by measuring the percentage of the area stained for glial fibrillary acidic protein (GFAP) and its immunofluorescence intensity. The results showed that AP treatment largely reduced astrocyte activation in both the hippocampus and the cortex (Figures 3C and 3D). Western blot analysis of brain cortical tissue further revealed that Iba1 and GFAP protein levels were significantly elevated in 5×FAD mice compared with WT controls, and AP administration led to a marked reduction in the expression of these proteins in 5×FAD mice (Figures 3E and 3F), underscoring the anti-neuroinflammatory effects of AP. Additional analysis of inflammatory markers, including *Il1a*, *Il1b*, *Il6*, *Tnfa*, and *Cxcl1*, revealed significant upregulation of their expression in 5×FAD mice compared with WT controls, and AP treatment also substantially decreased the mRNA levels of these inflammatory factors in 5×FAD mice (Figure 3G). These findings indicate that AP treatment reduces gliosis and may provide neuroprotection by suppressing neuroinflammation.

### AP treatment does not affect the density of neurons in the brains of 5×FAD mice

Given that Aβ can lead to neuronal death and dysfunction,^28^^,^^29^ we assessed neuronal health in both 5×FAD and WT mice. Immunofluorescence analysis revealed a trend toward a reduction in the number of neurons in the hippocampus of 5×FAD mice compared with WT mice. However, the administration of AP appeared to improve this trend, although the results were not statistically significant (Figures S3A and S3B). We further used Nissl staining to examine the distribution of neurons in the hippocampus and cortex. We found a trend toward fewer hippocampal neurons in 5×FAD mice relative to WT mice, with AP administration resulting in an apparent improvement in this trend. Still, the difference was not statistically significant (Figures S3C and S3D). To further investigate the potential impact of AP on cortical neurons, we extracted protein from the cerebral cortex tissues of three distinct groups of mice to assess alterations in neuron-associated proteins. Compared with the tissue samples from WT mice, NeuN protein expression was marginally lower in samples from 5×FAD mice. In contrast, the level of NeuN protein in tissue samples from the AP-treated group did not change significantly (Figure S3E). These findings suggest that AP treatment does not exert a substantial effect on neuronal density in the brains of 5×FAD mice.

### AP treatment suppresses the cerebral cholesterol accumulation in 5×FAD mice

**Figure 4:**
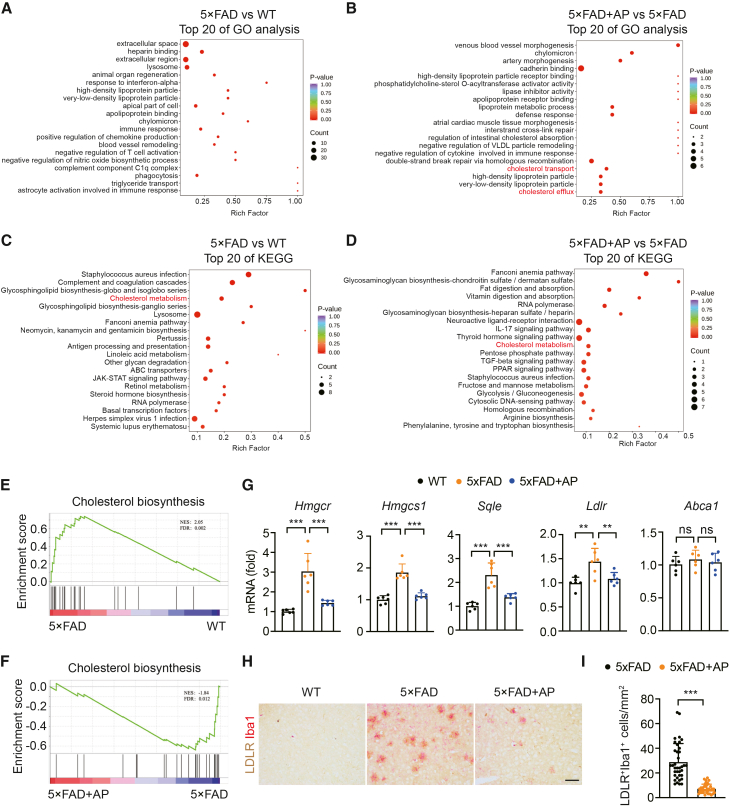
AP suppresses cerebral cholesterol accumulation (A and B) Bubble diagram of GO analysis of differentially expressed proteins. (C and D) Bubble diagram of KEGG analysis of differentially expressed proteins. (E and F) GSEA enrichment analysis diagram of genes related to cholesterol synthesis. (G) Quantitative PCR analysis of genes associated with cholesterol synthesis and cholesterol flux in the cortex tissue (*N* = 6). Each data point represents the test result of one mouse. (H) Immunohistochemistry staining with anti-Iba1 and anti-LDLR antibodies in the cortex. Scale bar, 100 μm. Sections were collected at approximately Bregma −1.70 mm (or immediately adjacent levels). (I) Quantitative immunohistochemistry staining of Iba1 and LDLR (5×FAD: *N* = 6, n = 6–7; 5×FAD+AP: *N* = 6, n = 6–7). Each data point represents the test result of one tissue slice. Data are expressed as mean ± SEM; *N* = the number of mice, *n* = number of fields of view per section from a single mouse. ANOVA followed by Bonferroni post hoc test was used. Statistical significance is indicated as ns: not significant, ∗∗*p* < 0.01, ∗∗∗*p* < 0.001.

To investigate the potential mechanisms by which AP mitigates cognitive impairment and inhibits microglial senescence in 5×FAD mice, fresh cerebral cortex tissues were collected from three experimental groups for proteomic analysis (data were stored in a public database: https://www.iprox.cn/page/project.html?id=IPX0014134000). As shown in Figure S4A, 212 upregulated and 126 downregulated proteins were identified in the 5×FAD group compared with the WT group. Compared with the 5×FAD group, AP treatment led to the upregulation of 142 proteins and the downregulation of 115 proteins. The gene ontology (GO) enrichment analysis bubble chart of differentially expressed proteins revealed that, in comparison with those in the WT group, proteins associated with lipoproteins were upregulated in the 5×FAD group (Figure 4A). However, these proteins, as well as cholesterol transporters, were downregulated following AP administration (Figure 4B). The bubble chart from the Kyoto Encyclopedia of Genes and Genomes (KEGG) enrichment analysis of the differentially expressed proteins showed that the impact of AP was associated with the regulation of the cholesterol metabolism pathway (Figures 4C and 4D). Gene set enrichment analysis (GSEA) demonstrated that, compared with the WT group, proteins related to cholesterol synthesis were significantly upregulated in the 5×FAD group, whereas they were markedly downregulated following AP administration (Figures 4E and 4F). Furthermore, RT‒qPCR validation analysis revealed that the expression of certain cholesterol synthesis-related genes, including hydroxymethylglutaryl-CoA reductase (*Hmgcr*), hydroxymethylglutaryl-CoA synthase (*Hmgcs1*), and squalene monooxygenase (*Sqle*), was elevated in the 5×FAD group compared to those in the WT group, but reduced after AP treatment (Figure 4G).

Cholesterol synthesis in the brain predominantly occurs in neurons via a *de novo* synthesis pathway, with cholesterol subsequently being secreted. We investigated the impact of AP on cholesterol dynamics within the brains of 5×FAD mice. RT-qPCR analysis revealed an increase in the cholesterol influx gene, low-density lipoprotein receptor (*Ldlr*), which was reduced in the group treated with AP (Figure 4G). Conversely, the expression of the cholesterol efflux gene ATP-binding cassette transporter A1 (*Abca1*) was downregulated, but increased following AP treatment (Figure 4G). Furthermore, we examined the influence of AP on cholesterol levels in microglia within the brains of 5×FAD mice. Immunohistochemical staining revealed a significant increase in the number of microglia expressing LDLR in 5×FAD mice compared with WT mice, which was significantly reduced after AP administration (Figures 4H and 4I). Together, these findings suggest that microglial senescence in the cerebral cortex of 5×FAD mice may be driven by cholesterol accumulation, and that AP effectively mitigates intracellular cholesterol accumulation.

### AP treatment reduces LDL-induced cholesterol accumulation and cell senescence in BV2 cells

**Figure 5:**
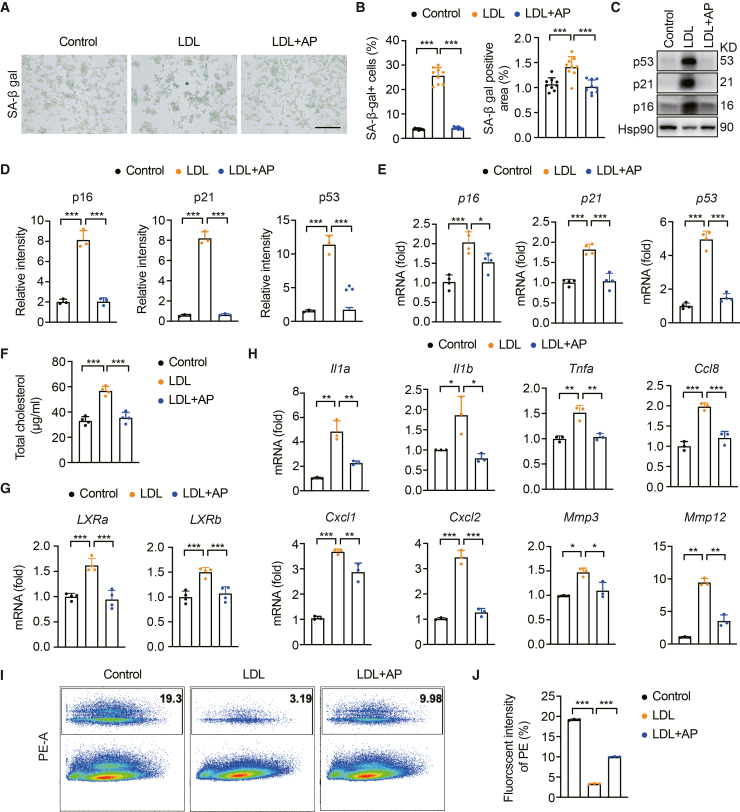
AP reduces LDL-induced cholesterol accumulation and cellular senescence in BV2 cells (A) SA-β-gal staining of BV2 cells induced by LDL. Scale bar, 150 μm. (B) Quantitative LDL-induced SA-β-gal staining (*N* = 3, *n* = 3). Each data point represents the test result of one field of view. (C) Western blot analysis of p16, p21, and p53 expression in LDL-induced BV2 cells (*n* = 3). Each data point represents the test result of one sample and similar results were repeated in three biologically independent experiments. (D) Quantitative data of p16, p21, and p53 protein levels (*N* = 3). Each data point represents the test result of one sample. (E) Quantitative PCR analysis of senescence-associated genes p16, p21, and p53 in LDL-induced BV2 cells (*N* = 4). Each data point represents the test result of one sample. (F) Determination of cholesterol content in LDL-induced BV2 cells (*N* = 4). Each data point represents the test result of one sample. (G) Quantitative PCR analysis of cholesterol sensor-related genes LXRα and LXRβ in LDL-induced BV2 cells (*N* = 4). Each data point represents the test result of one sample. (H) Quantitative PCR analysis of SASP factors in LDL-induced BV2 cells (*N* = 3). Each data point represents the test result of one sample. (I) Flow cytometry analysis of LDL-induced phagocytic function in BV2 cells (*N* = 3). Each data point represents the test result of one sample. (J) Quantitative data of flow cytometry (*N* = 3). Each data point represents the test result of one sample and similar results were repeated in three biologically independent experiments. Data are expressed as mean ± SEM. *N* = number of independent experiments, *n* = number of fields of view analyzed for each experiment. ANOVA followed by Bonferroni post hoc test was used. Statistical significance is indicated as ∗*p* < 0.05, ∗∗*p* < 0.01, ∗∗∗*p* < 0.001.

Based on the previous observation, we further investigated the potential mechanism by which AP affects cholesterol accumulation in microglial cells using BV2 cells *in vitro*. We first evaluated the cytotoxic effects of AP on BV2 microglial cells. AP concentrations exceeding 10 μM significantly reduced cell viability (Figure S5A). To investigate the impact and underlying mechanism of AP on cholesterol accumulation and cellular senescence, we performed co-culture experiments with HT22 neuronal cells and BV2 microglial cells in the presence and absence of AP treatment. The results showed that microglia did not exhibit a senescent phenotype under both conditions (Figures S5B and S5C). Next, we used an *in vitro* LDL-induced BV2 cell model and analyzed senescence-related markers. We found that the LDL-treated group showed a modest increase in the proportion of SA-β-gal-positive areas compared with the control group, and AP treatment significantly decreased both cell number and the percentage of positive cells in the LDL-treated group (Figures 5A and 5B). Similarly, Western blot and RT-qPCR analyses revealed that the expression levels of certain senescence-associated proteins and genes, such as p16, p21, and p53, were elevated in the LDL group compared with those in the control group, whereas AP treatment led to a reduction in their expression levels (Figures 5C‒5E). The cholesterol content in BV2 cells post-LDL treatment was quantified using a cholesterol detection kit. The results revealed a significant increase in cholesterol content in the LDL-treated group compared with the control group, which was also substantially decreased following AP treatment (Figure 5F). RT-qPCR analysis revealed that, compared with the control group, the LDL-treated group presented an elevated expression of cholesterol receptor-related genes, including *LXRα* and *LXRb*, which was subsequently reduced following AP treatment (Figure 5G). RT-qPCR analysis revealed that the expression of certain inflammatory factors was significantly elevated in the LDL group compared with the control group, and this increased expression was significantly reduced following AP treatment (Figure 5H). Flow cytometry analyses demonstrated that the phagocytic function of BV2 cells was significantly compromised in the LDL-treated group compared with the control group. Once treated with AP, it partially restored their phagocytic capacity (Figures 5I and 5J). These findings suggest that AP has the potential to mitigate LDL-induced cholesterol accumulation and cellular senescence in BV2 cells.

### AP treatment inhibits STAT3 activity in the brains of 5×FAD mice and LDL-treated BV2 cells

**Figure 6:**
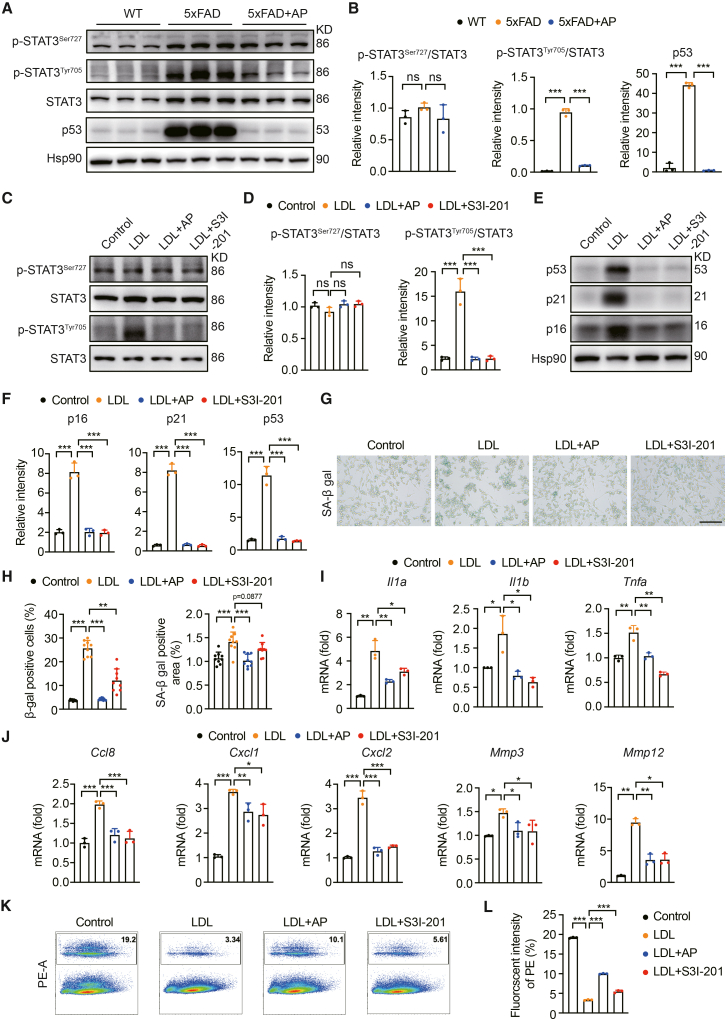
AP inhibits STAT3 activation in both 5×FAD mice and BV2 cells (A) Western blot analysis of p53 and STAT3 expression in the cortex of 5×FAD mice. Each data lane represents the test result of one mouse. (B) Quantitative data of p53 and STAT3 protein levels (*N* = 3). Each data point represents the test result of one mouse. (C) Western blot analysis of STAT3 expression in LDL-induced BV2 cells. Each data point represents the test result of one sample and similar results were repeated in three biologically independent experiments. (D) Quantitative data of STAT3 protein levels (*N* = 3). Each data point represents the test result of one sample. (E) Western blot analysis of p16, p21, and p53 expression in LDL-induced BV2 cells. Each data point represents the test result of one sample and similar results were repeated in three biologically independent experiments. (F) Quantitative data of p16, p21, and p53 protein levels (*N* = 3). Each data point represents the test result of one sample. (G) SA-β-gal staining of BV2 cells induced by LDL. Scale bar, 150 μm (*N* = 3, *n* = 3). (H) Quantitative LDL-induced SA-β-gal staining (*N* = 3, *n* = 3). Each data point represents the test result of one field of view. (I and J) Quantitative PCR analysis of SASP factors in LDL-induced BV2 cells (*N* = 3). Each data point represents the test result of one sample. (K) Flow cytometry analysis of LDL-induced phagocytic function in BV2 cells (*N* = 3). Each data point represents the test result of one sample. (L) Quantitative analysis of flow cytometry (*N* = 3). Each data point represents the test result of one sample and similar results were repeated in three biologically independent experiments. Data are expressed as mean ± SEM; *N* = number of mice or independent experiments, *n* = number of fields of view analyzed for each experiment. ANOVA followed by Bonferroni post hoc test was used. Statistical significance is indicated as ns: not significant, ∗*p* < 0.05, ∗∗*p* < 0.01, ∗∗∗*p* < 0.001.

Previous studies have demonstrated that LDL or oxidized low-density lipoprotein (ox-LDL) induces substantial activation of STAT3. This transcription factor mediates the effects of cytokines and growth factors and regulates target gene expression, leading to cell proliferation, survival, and immune responses.^30^^,^^31^ We observed a notable increase in p-STAT3Tyr705 protein levels in the 5×FAD mouse tissue samples, which were reduced by AP treatment (Figures 6A and 6B). Notably, the ability of AP to suppress STAT3 activation by reducing the phosphorylation level of STAT3 at the Tyr705 site in LDL-treated BV2 cells mirrored the effects of the STAT3 inhibitor S3I-201 (Figures 6C and 6D). Furthermore, we performed an overlap analysis to identify the shared genes linked to AP from the PharmMapper server and those associated with Aβ, aging, Alzheimer’s disease, and cholesterol metabolism from the GeneCards database. It yielded 15 common genes, which were shown in a Venn diagram (Figure S4B). A protein‒protein interaction (PPI) analysis of these 15 genes was then conducted using the STRING database, and the results were visualized via Cytoscape V3.9.1 (Figure S4C). Aging-related IL-6 cytokines induce self-reinforcing and cross-reinforcing senescence and inflammatory microenvironments.^32^ MMP9 has been associated with osteoclastogenesis,^33^ while MAPK14 promotes chondrocyte senescence.^34^ Based on the analysis of these 15 genes, we reasoned that AP could exhibit a stronger association with the IL-6 pathway. Molecular docking analysis also revealed a strong interaction between AP and IL-6 (Figure S4D). Many studies have underscored the critical role of the IL-6/STAT3 signaling pathway in aging processes.^35^^,^^36^ This network pharmacology analysis further suggested that AP might target the IL-6/STAT3 signaling pathway to cf. therapeutic benefits for age-related neurodegenerative disorders.

Previous research has demonstrated that STAT3 phosphorylation activates p53, which can induce cellular senescence through the p53/p21 signaling pathway.^37^^,^^38^ Western blot analysis revealed significant upregulation of p53 expression in the tissue samples from 5×FAD mice compared with those from WT mice (Figures 6A and 6B). In contrast, the administration of AP resulted in a marked reduction in p53 protein levels in the tissue samples from 5×FAD mice (Figures 6A and 6B). To further investigate the protective role of AP against BV2 cell senescence, we compared the effects of AP treatment with those of the STAT3 inhibitor S3I-201. BV2 cells were exposed to exogenously supplemented LDL to induce cellular senescence. As expected, treatment with AP significantly delayed LDL-induced cellular senescence, similar to S3I-201, as evidenced by the reduced expression of p53/p21 and p16 (Figures 6E and 6F), decreased SA-β-gal activity (Figures 6G and 6H), and lower levels of SASP factors (Figures 6I and 6J). Flow cytometry analysis further demonstrated that the compromised phagocytic function of LDL-treated BV2 cells was substantially restored by AP treatment and by S3I-201 (Figures 6K and 6L). These findings suggest that the ability of AP to mitigate LDL-induced senescence in BV2 cells may be through its action on STAT3 activation.

### AP attenuates BV2 cell senescence by suppressing STAT3 activation

**Figure 7:**
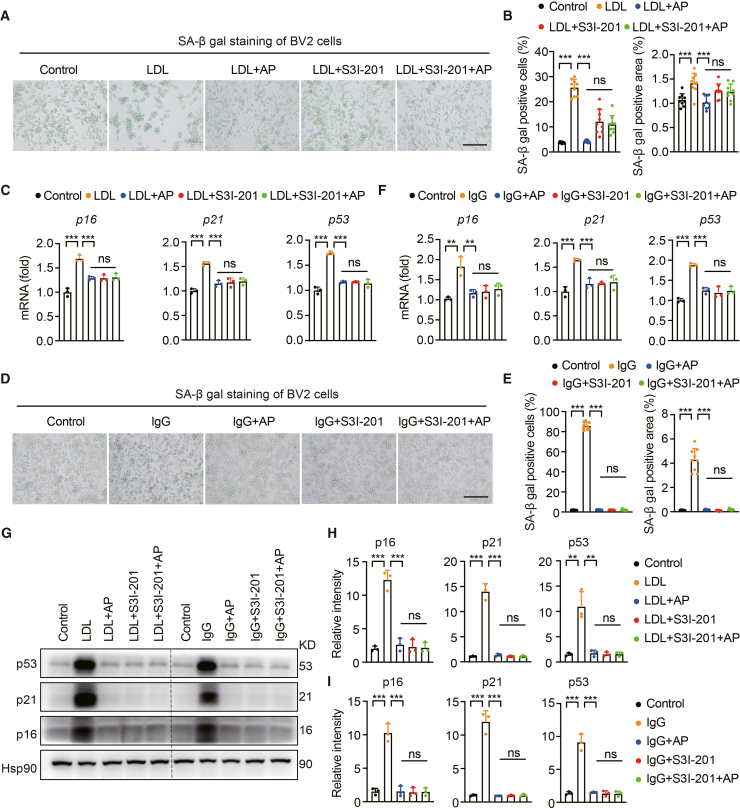
AP inhibits the cell aging process of BV2 cells by suppressing the activity of STAT3 (A) SA-β-gal staining of BV2 cells induced by LDL. Scale bar, 150 μm. (B) Quantitative SA-β-gal staining (*N* = 3, *n* = 3). Each data point represents the test result of one field of view. (C) Quantitative PCR analysis of senescence-associated genes p16, p21, and p53 in LDL-induced BV2 cells (*N* = 3). Each data point represents the test result of one sample. (D) SA-β-gal staining of BV2 cells induced by IgG. Scale bar, 150 μm. (E) Quantitative SA-β-gal staining (*N* = 3, *n* = 3). Each data point represents the test result of one field of view. (F) Quantitative PCR analysis of senescence-associated genes p16, p21, and p53 in IgG-induced BV2 cells (*N* = 3). Each data lane represents the test result of one sample. (G) Western blot analysis of p16, p21, and p53 expression in BV2 cells. Each data lane represents the test result of one sample and similar results were repeated in three biologically independent experiments. (H) Quantitative data of p16, p21, and p53 protein levels in LDL-induced BV2 cells (*N* = 3). Each data lane represents the test result of one sample. (I) Quantitative data of p16, p21, and p53 protein levels in IgG-induced BV2 cells (*N* = 3). Data are expressed as mean ± SEM. N = number of independent experiments, n = number of fields of view analyzed for each experiment. ANOVA followed by Bonferroni post hoc test was used. Statistical significance is indicated as ∗∗*p* < 0.01, ∗∗∗*p* < 0.001.

We next investigated whether AP mitigates cellular senescence by modulating STAT3 activation. Our results demonstrated that either S3I-201 or AP alone reduced SA-β-gal activity and the expression of senescence-related genes in LDL-treated BV2 cells. However, when S3I-201 blocked STAT3 activation, AP failed to suppress senescence in these cells further (Figures 7A‒7C), indicating that the anti-senescence effect of AP in BV2 cells is relying on STAT3 activation.

Recent studies have shown that IgG induces cellular senescence across multiple cell types.^39^ We found that AP treatment largely alleviated increased SA-β-gal activity, upregulated inflammatory markers, and partially compromised phagocytic function in IgG-treated BV2 cells (Figures S6A‒S6F). S3I-201 presented similar roles as AP (Figure S6). Notably, compared with AP treatment alone, the combination of AP and S3I-201 did not significantly enhance the suppression of senescence markers or the reduction in elevated levels of aging-related proteins (p16, p21, and p53) in BV2 cells (Figures 7D‒7I). These results indicate that AP likely exerts its anti-senescence effects through a STAT3-mediated pathway, as STAT3 inhibition with S3I-201 negates AP’s protective effects against BV2 cell senescence.

## Discussion

Aging is the primary risk factor for AD.^40^ Cellular senescence is increasingly recognized as a key contributor to AD.^41^^,^^42^ Previous studies have detected senescent astrocytes, microglia, endothelial cells, and neurons in the brains of AD patients and relevant animal models.^43^^,^^44^^,^^45^^,^^46^^,^^47^^,^^48^^,^^49^ Removing these senescent cells through genetic or pharmacological approaches has been shown to reduce Aβ peptide levels and tau-related neuropathology, and to ameliorate memory deficits in AD mouse models.^50^^,^^51^^,^^52^ These findings highlight that cellular senescence is a significant driver of AD pathology and identifying the drugs that can target cellular senescence in CNS cells would be a promising strategy for AD treatment. While previous studies have indicated the beneficial effects of AP,^53^^,^^54^^,^^55^ they have primarily focused on its anti-inflammatory mechanisms. In this study, we reveal that AP may also modulate brain cell aging in AD. We have identified that AP is critical in alleviating microglial senescence and ameliorating AD pathology. In both LDL- and IgG-induced senescent BV2 microglia, AP exerts a protective effect by reducing the senescent phenotype. AP can also mitigate microglial senescence in 5×FAD mice, a well-established AD model, potentially by alleviating inflammation and AD pathology, highlighting its therapeutic potential.

Aβ deposition triggers microglial activation, promoting the release of proinflammatory cytokines that exaggerate neuroinflammation.^56^ In this study, AP administration markedly improves behavioral outcomes by decreasing Aβ accumulation and inflammation in 5×FAD mice. In addition, previous research indicates that AD is characterized by microglial overgrowth and abnormal morphology, which coincide with the buildup of the Aβ and tau proteins.^57^ Astrocyte proliferation plays a key role in driving inflammation, often signaled by increased GFAP expression, a well-established indicator of gliosis.^56^ Our study demonstrates that AP treatment significantly reduces Iba1 and GFAP levels in 5×FAD mice, while also lowering proinflammatory cytokine levels, thereby confirming the potent anti-inflammatory effects of AP.

Genome-wide association studies and expression studies have revealed that roughly one-third of the genes associated with AD risk are involved in sterol and lipid metabolism.^58^ Evidence from genetics, epidemiology, and biochemistry suggests that cholesterol homeostasis is disrupted in AD and that AD susceptibility might stem from changes in cholesterol metabolism.^59^^,^^60^ GO and KEGG pathway enrichment functional analyses based on proteomic analysis reveal that high-density lipoprotein particles, very-low-density lipoprotein particles, and cholesterol metabolism are implicated in the AP-treated 5×FAD group compared with the untreated 5×FAD group. RT-qPCR analysis confirms that AP treatment leads to the downregulation of HMGCR, HMGCS1, SQLE, and LDLR in 5×FAD mice. Furthermore, compared with the untreated 5×FAD group, LDLR protein expression in the 5×FAD group treated with AP is lower. Based on these findings, our study demonstrates reduced cholesterol accumulation in the 5×FAD group subjected to AP treatment. We have established a senescent cell model by treating BV2 cells with LDL and verified the protective effect of AP against LDL-induced cellular senescence.

IL-6 is a multifaceted cytokine pivotal for regulating cell growth, survival, and differentiation, and its dysregulation has been implicated in numerous diseases, including inflammatory conditions and cancers.^61^ Prolonged radiation exposure can trigger DNA damage response in healthy fibroblasts, promoting IL-6 secretion, which contributes to cellular senescence,^35^ suggesting a link between IL-6 and aging-related processes, particularly in the context of cellular stress. Using a network pharmacology approach, 15 key hub genes associated with AP, aging, Aβ, cholesterol metabolism, and AD have been identified in this study. Among these genes, IL-6 and TNF are identified as critical inflammatory factors, highlighting their roles in driving inflammation across these conditions. Molecular docking studies have further revealed a potential interaction between AP and IL-6, suggesting that IL-6 signaling may be involved in AP’s protective role in AD.

In various cell types, IL-6 signaling is primarily mediated by STAT3, a transcription factor central to cellular regulation and intercellular communication.^62^^,^^63^^,^^64^ The activation of STAT3 involves phosphorylation at key tyrosine and serine residues, particularly tyrosine 705, which drives STAT3 homodimerization.^65^ This dimerization enables STAT3 to translocate to the nucleus, where it regulates the transcription of genes involved in processes such as inflammation.^66^ In addition to its role in inflammation, STAT3 plays a significant role in oxidative stress-induced senescence, making it a critical mediator of aging-related processes.^67^ In the cerebral cortex of 5×FAD mice, AP treatment significantly reduces STAT3 activation. AP not only inhibits STAT3 activation but also markedly decreases the number of senescent cells and the expression of associated inflammatory factors in an *in vitro* model of LDL-induced BV2 cell senescence. Furthermore, AP enhances the phagocytic capacity of BV2 cells, indicating the restoration of microglial functionality. Treatment with S3I-201, a presumed STAT3 inhibitor, closely mimics the effects of AP in an LDL/IgG-induced BV2 cell senescence model, suggesting that the anti-senescent effects of AP are likely mediated by STAT3 inhibition. Notably, the combined application of AP and S3I-201 does not yield a further inhibitory effect on cellular senescence beyond that of the individual treatments. This lack of additive or synergistic effects suggests that AP and S3I-201 might target the same or overlapping pathways, with STAT3 inhibition being a central mechanism.

Our research has revealed that AP ameliorates AD by increasing microglial phagocytic activity, reducing Aβ plaque accumulation, and enhancing cognitive performance, underscoring its potential as a therapeutic agent for AD. By inhibiting STAT3-mediated pathways, AP reduces microglial senescence and inflammation while restoring microglial functionality, indicating a mechanism through which AP mitigates inflammatory and senescent processes in 5×FAD mice.

### Limitations of the study

Although this study has identified a crucial anti-aging role for AP in the treatment of AD, several limitations remain. Firstly, whether the amelioration of behavioral deficits by AP in 5×FAD mice primarily resulting from the reduced senescent microglia remains to be determined. Selective ablation of senescent cells or targeted interventions against microglia would be better to elucidate this pivotal question in future study. Secondly, although we propose the STAT3 pathway as a potential molecular mechanism for mitigating cholesterol accumulation-induced microglial senescence, *in vivo* validation using corresponding conditional knockout mouse models is lacking. Thirdly, the sample size in the MWM experiments is relatively small (*N* = 5–6 animals per group), with limited representation of each sex (*N* = 3 males and *N* = 3 females per group). Larger, adequately powered cohorts with balanced sex stratification will be essential in future studies to confirm and extend the present findings.

## Resource availability

### Lead contact

Requests for further information and resources should be directed to and will be fulfilled by the lead contact, Gonghua Huang (gonghua.huang@gdmu.edu.cn).

### Materials availability

This study did not generate new, unique reagents.

### Data and code availability

All datasets used in this study are available from the corresponding author upon reasonable request. The mass data used in this study have been deposited in the ProteomeXchange Consortium with the iProX partner repository. The dataset can be authorized using a PXD number: PXD070443. Code: This paper does not report original code. Any additional information required to reanalyze the data reported in this paper is available from the lead contact upon request.

## Acknowledgments

We thank Prof. Bo Peng (Fudan University) for kindly providing the 5×FAD mice. We thank Caimei Song and Jun Liu for help with animal colony management and other support. We thank Drs. Yanyan Wang and Saadullah Khattak for manuscript editing. We thank Dr. Kaiwu He for providing technical assistance and suggestions in the revision.

This work was supported by the 10.13039/501100001809National Natural Science Foundation of China (32341004) and 10.13039/501100021171Guangdong Basic and Applied Basic Research Foundation (2021B1515130004, 2023A1515012838, and 2024A1515012922).

## Author contributions

H.S. and M.Y. conducted the experiments, analyzed the data, and wrote the manuscript. S.W. helped with cell culture; W.Q., Q.D., and W.X. assisted with animal experiments; Y.C. and X.Z. assisted with immunoblotting; and C.O. and Y.Z. assisted with RT-PCR data analysis. X.L. and Y.Z. supplied materials and provided technical assistance. G.H. secured funding, designed the study, and was involved in project administration and critical review of the manuscript.

## Declaration of interests

The authors declare no competing interests.

## STAR★Methods

### Key resources table

**Table:** 

REAGENT or RESOURCE	SOURCE	IDENTIFIER
**Antibodies**		

Iba1	Abcam	ab178847; RRID:AB_2832244
GFAP	Proteintech	16825-1-AP; RRID: AB_2109646
Aβ42	Abcam	ab201060; RRID: AB_2818982
NeuN	Proteintech	66836-1-Ig; RRID: AB_2882179
p16^Ink4a^	Santa Cruz	SC1661; RRID: AB_628067
p21^Cip1^	Santa Cruz	SC6246; RRID: AB_628073
Goat anti-mouse AF488	Boster	BA1126; RRID: AB_2827694
Goat anti-mouse AF594	Boster	BA1141; RRID: AB_2941989
Goat anti-rabbit AF488	Boster	BA1127; RRID: AB_3713475
Goat anti-rabbit AF594	Boster	BA1142; RRID: AB_3095580
Dectin-1	Abcam	ab287961; RRID: AB_2877055
CD68	Cell Signaling Technology	#97778S; RRID: AB_2928056
LDLR	Proteintech	10785-1-AP; RRID: AB_2281164
Iba1	Abcam	ab283319; RRID: AB_2924797
6E10	Biolegend	RUO803014; RRID: AB_2734547
p53	Cell Signaling Technology	#2524; RRID: AB_331743
STAT3^Tyr705^	Cell Signaling Technology	#9145; RRID: AB_2491009
STAT3^Ser727^	Cell Signaling Technology	#94994; RRID: AB_2800239
APP	Cell Signaling Technology	#76600; RRID: AB_2925222
BACE1	Cell Signaling Technology	#5606; RRID: AB_11028291
β-Actin	Cell Signaling Technology	#4967; RRID: AB_2797972
Hsp90	Snta Cruz	SC13119; RRID: AB_675659
HRP-labeled Goat Anti-Rabbit IgG(H+L)	Beyotime	A0208; RRID: AB_2892644
HRP-labeled Goat Anti-mouse IgG(H+L)	Beyotime	A0216; RRID: AB_3696739

**Chemicals, peptides, and recombinant proteins**		

Andrographolide	Aladdin	CAS: 5508-58-7, Lot# F2209080
STAT3 inhibitor	Selleck	S7024
IgG	Bioss	bs-0296P
LDL	Yiyuan	YB-001

**Critical commercial assays**		

IHC kit	DAKEWE	4971011
Senescence β-Galactosidase Staining Kit	Sigma	CS0030-1KT
Cholesterol kit	Beyotime	S0211S
Aβ1-40 ELISA kit	Elabscience	E-EL-H0542
Aβ1-42 ELISA kit	Elabscience	E-EL-H0543

**Deposited data**		

Proteomics	This paper	PXD070443

**Experimental models: Cell lines**		

BV2 cell	National Collection of Authenticated Cell Cultures	SCSP-5208

**Software and algorithms**		

ImageJ	National Institute of Health	https://imagej.net/ij/
GraphPad Prism	GraphPad Software	https://www.graphpad.com/
FlowJo	BD Life Sciences	https://www.flowjo.com/

### Experimental model and study participant details

#### Animals

All the animal studies were approved by the Institutional Animal Care and Use Committee of Guangdong Medical University (Approval No. GDY2004002).

The 5×FAD mice were generously provided by Prof. Bo Peng (Fudan University), bred, and maintained on the C57BL/6J background under specific pathogen-free conditions. Both male and female mice were used in the experiments, with littermates serving as controls. Genotyping was performed using genomic DNA extracted from tail snips. Mice were housed under a 12-hour light/12-hour dark cycle, with access to food and water *ad libitum*. At 7 months of age, wild-type (WT) and 5×FAD mice were randomly assigned to either the AP treatment or control groups. AP (CAS: 5508-58-7, Lot# F2209080, Aladdin, Shanghai, China), with a purity of 98%, was administered intraperitoneally at a dose of 2.0 mg/kg, three times per week for 8 consecutive weeks. WT and 5×FAD mice in the control groups received saline as a vehicle. In all animal and cell experiments involved in this paper, AP was dissolved and diluted in 0.9% normal saline. All animal experimental procedures were approved by the Experimental Animal Center of Guangdong Medical University.

#### Cell culture

The BV2 microglial cell line was obtained from the Type Culture Collection of the Chinese Academy of Sciences. Cells were maintained in Dulbecco’s Modified Eagle Medium (DMEM; Gibco, 6125017) supplemented with 10% fetal bovine serum (FBS; Gibco, 10091148) and 1% penicillin-streptomycin-glutamine (PSG; Gibco, 10378-016). Cultures were incubated at 37°C in a humidified atmosphere containing 5% CO_2_. BV2 cells were treated with 5 μM AP in the presence of either IgG (1.2 mg/mL, Bioss, bs-0296P) or LDL (100 μg/mL, Yiyuan, YB-001) for 24 hours. Following treatment, cells were collected and processed for subsequent analyses.

### Method details

#### Immunofluorescence (IF)

Mice were anesthetized and transcardially perfused with ice-cold phosphate-buffered saline (PBS), and brains were subsequently harvested. The brains were fixed overnight at 4°C in 4% paraformaldehyde. After fixation, they were submerged in a 40% sucrose solution at 4°C with gentle shaking until they sank. Coronal sections (20 μm) were obtained using a cryostat (Thermo Fisher). The sections were incubated in blocking/permeabilization buffer (5% fetal bovine serum and 0.3% Triton X-100 in PBS) at room temperature for 1 hour. Subsequently, they were incubated overnight at 4°C with primary antibodies, including Iba1 (ab178847, Abcam, 1:100), GFAP (16825-1-AP, Proteintech, 1:200), Aβ42 (ab201060, Abcam, 1:50), NeuN (66836-1-Ig, Proteintech, 1:200), p16^Ink4a^ (SC1661, Santa Cruz, 1:50), and p21^Cip1^ (SC6246, Santa Cruz, 1:50). After incubation with primary antibodies, the sections were incubated with secondary antibodies conjugated to Alexa Fluor 488 or 594. The sections were then treated with an anti-fade reagent to prevent fluorescence quenching and coverslipped. Images were acquired using a Leica SP8 confocal microscope and analyzed with ImageJ. IF data were collected from five mice per group. For each mouse, three sections were selected, with one area assessed on each section. The data were obtained from the cerebral cortex and hippocampus.

#### Immunohistochemistry (IHC)

Free-floating cryosections (20 μm thick) were collected in PBS. Immunohistochemistry was performed on brain sections collected at approximately Bregma -1.70 mm (or immediately adjacent levels) following the kit instructions (4971011, DAKEWE). Briefly, brain sections were incubated with 3% H_2_O_2_ for 12 minutes to block endogenous peroxidase activity. After blocking with a solution containing 5% goat serum and 3% Triton X-100 in PBS at 37°C for 30 minutes, sections were incubated overnight at 4°C with primary antibodies, including Iba1 (ab178847, Abcam, 1:500), Dectin-1 (ab287961, Abcam, 1:500), CD68 (#97778S, Cell Signaling Technology, 1:100), LDLR (10785-1-AP, Proteintech, 1:100), Iba1 (ab283319, Abcam, 1:500), p16^Ink4a^ (SC1661, Santa Cruz, 1:10), 6E10 (RUO803014, Biolegend, 1:200) and p21^Cip1^ (SC6246, Santa Cruz, 1:10). Following thorough washing in PBS, sections were incubated with HRP-conjugated secondary antibodies at room temperature for 1 hour. The signal was developed using a DAB substrate according to standard protocols. Subsequently, sections were dehydrated through a graded ethanol series, cleared in xylene, and mounted with coverslips. Bright-field images were acquired using a Leica microscope. IHC data were collected from five mice per group. For each mouse, three sections were selected, with one area assessed on each section. The data were obtained from the cerebral cortex.

#### Morris water maze (MWM) test

The MWM test was conducted to evaluate spatial learning and memory. A hidden platform was submerged 0.5 cm below the water surface. Mice underwent three consecutive days of training. Each training lasted a maximum of 60 seconds; if a mouse failed to locate the platform within this time, it was gently guided to it and allowed to remain there for 10 seconds. During each trial, mice were released from different quadrants, facing the pool wall, into a circular pool maintained at 22 ± 1°C and rendered opaque with non-toxic white paint. On the last day, a probe trial was conducted in which the platform was removed, and mice were allowed to swim freely for 60 seconds to assess their spatial memory. All trials were recorded and analyzed using a computerized video tracking system.

#### SA-β-gal staining

SA-β-gal staining was conducted to detect senescent cells by measuring β-galactosidase activity at pH 6.0, using the Senescence β-Galactosidase Staining Kit (CS0030-1KT, Sigma). Brain sections or BV2 cells were rinsed in PBS and fixed with 4% paraformaldehyde for 15 minutes at room temperature. Following fixation, samples were washed thoroughly with PBS and incubated with freshly prepared SA-β-gal staining solution, which contained X-gal (1 mg/mL). Samples were incubated in a dry incubator at 37°C without CO_2_ for 12-16 hours. After incubation, sections were washed again with PBS, and senescent cells exhibiting blue staining were visualized under a light microscope. Images were captured using a Leica DM500 bright-field microscope. Quantification of SA-β-gal-positive areas was performed in representative fields using ImageJ software. The brain sections data were collected from five mice per group. For each mouse, three sections were selected, with one area assessed on each section. The data were obtained from the cerebral cortex.

#### Proteomics

The mice were euthanized by carbon dioxide asphyxiation, following which they were washed with 15 ml of 1×PBS pre-cooled solution by ventricular perfusion. The brain was dissected out, with the right hemisphere being fixed with 4% paraformaldehyde overnight 4°C, and the left hemisphere being quickly sectioned to obtain samples of cerebral cortex tissue. These samples were then frozen in liquid nitrogen and stored at -80°C. The extraction, detection, and quantitative analysis of protein profiling in the samples were performed by Wuhan Metware Biotechnology Co., Ltd. (www.metware.cn). Detailed methods are provided in the following:

Samples were retrieved from the -80°C freezer and ground into powder using liquid nitrogen. Subsequently, an appropriate amount of the powder is transferred into a 1.5 mL centrifuge tube, followed by the addition of lysis buffer (containing 8 M urea, 1 mM PMSF, and 2 mM EDTA), performed ultrasonic lysis for 5 minutes on ice, and centrifuged at 15,000 × *g* at 4°C for 10 minutes to collect the supernatant. Finally, determined the protein concentration using a BCA assay kit.

Protein (100 mg) was taken based on the measured protein concentration and the volume was adjusted to 200 μL with 8 M urea. The sample was then reduced with 5 mM DTT at 37 °C for 45 min and alkylated with 11 mM iodoacetamide (IAM) in the dark at room temperature for 15 min. Subsequently, 800 μL of 25 mM ammonium bicarbonate solution and 3 μl of trypsin (Promega) were added, followed by overnight digestion at 37°C. The pH of the digested peptides was adjusted to 2-3 using 20% TFA, and the peptides were desalted using C18 resin (Millipore, Billerica, MA). Finally, the peptide concentration was determined using the Pierce Quantitative Colorimetric Peptide Assay Kit with standards (Thermo Fisher Scientific).

Samples were separated using the Vanquish Neo UHPLC liquid chromatography system. The mobile phase A consisted of 0.1% formic acid aqueous solution, while mobile phase B was acetonitrile containing 0.1% formic acid. The injection mode employed a trap-and-elute dual-column method, with a PepMap Neo Trap Cartridge (300 μm × 5 mm, 5 μm) as the trapping column and an Easy-SprayTM PepMapTM Neo UHPLC column (150 μm × 15 cm, 2 μm) as the analytical column. The column temperature was controlled at 55°C, with an injection amount of 200 ng, a flow rate of 2.5 μl/min, an effective gradient of 6.9 minutes, and a total runtime of 8 minutes.

DIA analysis utilized the Vanquish Neo system (Thermo Fisher Scientific) for chromatographic separation. Samples separated by nano-flow high-performance liquid chromatography were subjected to DIA (Data-Independent Acquisition) mass spectrometry analysis using the Orbitrap Astral high-resolution mass spectrometer (Thermo Scientific). The detection mode was positive ion mode, with a precursor ion scan range of 380-980 m/z, a primary mass resolution of 240000 at 200 m/z, a Normalized AGC Target of 500%, and a Maximum IT of 5 ms. MS2 was performed using DIA data acquisition mode, with 299 scan windows, an Isolation Window of 2 Th, HCD Collision Energy of 25%, a Normalized AGC Target of 500%, and a Maximum IT of 3 ms.

#### Network pharmacology analyses

The PubChem database (https://pubchem.ncbi.nlm.nih.gov) was used to search for the chemical structure and SMILES notation of AP and save it as an SDF format file. Then, its potential protein targets were acquired from the SwissTargetPrediction (http://swisstargetprediction.ch/). Targets related to aging, Aβ, AD, and cholesterol metabolism were filtered from GeneCards (https://www.genecards.org/), OMIM (https://www.omim.org/), MalaCards (https://www.malacards.org/), and TTD (http://db.idrblab.net/ttd/) databases. All the collected targets were validated by searching and selecting species as “Homo sapiens” in the Uniprot database (https://www.uniprot.org/). The obtained targets were imported into the STRING database (htt ps://string-db.org), with the species limited to “Homo sapiens” and the minimum interaction score set to highest confidence level of 0.9. Subsequently, the TSV file downloaded from the STRING database was subjected to visualization analysis in Cytoscape software.

#### Molecular docking

The three-dimensional (3D) protein structures of key targets were downloaded from the Protein Data Bank (PDB; http://www.rcsb.org) in PDB format. Additionally, for proteins with incomplete structural data, homology modeling was performed using the automatic protein structure homology modeling server SwissModel (https://swissmodel.expasy.org) to complete the missing loops and domains. The 3D structures of the top 10 active compounds ranked by degree centrality were downloaded from PubChem and saved in PDB format. Molecular docking of these active compounds with protein targets was implemented using Autodock Tools 1.5.6 and Autodock Vina 1.1.2, with the center of the docking box defined as the coordinates of the original ligand within the receptor structure. The molecular docking results were visualized using PyMol 2.4.0.

#### Determination of cholesterol content

Cholesterol levels were measured using a commercial assay kit (S0211S, Beyotime) according to the manufacturer's instructions. BV2 cells treated with LDL, with or without AP, were harvested and lysed using BeyoLysis™ Buffer A for Metabolic Assay. The cell suspension was homogenized on ice, followed by centrifugation at 12,000 × g for 5 minutes at 4°C. The resulting supernatant was collected and subsequently analyzed using a microplate reader (1550, Thermo Fisher).

#### ELISA

Whole brain cortex tissue was homogenized in protein lysis buffer (RIPA; P0013B, Beyotime) supplemented with the mixture of protease and phosphatase inhibitors (P1045, Beyotime) and PMSF (ST2573, Beyotime). Protein supernatant was determined using the Aβ1-40 (E-EL-H0542, Elabscience) and Aβ1-42 ELISA kit (E-EL-H0543, Elabscience). The data were obtained from 6 mice per group.

#### Western blot

Whole brain cortex tissue or cultured BV2 cells were homogenized in protein lysis buffer (RIPA; P0013B, Beyotime) supplemented with the mixture of protease and phosphatase inhibitors (P1045, Beyotime) and PMSF (ST2573, Beyotime). Protein concentration was determined using the Pierce BCA protein assay kit (23227, Thermo Pierce). 20 μg total protein was separated by SDS-PAGE and transferred to polyvinylidene difluoride (PVDF; IPVH00010, Millipore) membranes. After incubating in blocking solution with 3% bone serum albumin (BSA; A8020, Solaribio) in PBST at room temperature for 1.5 hours, membranes were incubated with primary antibodies including Iba1 (ab178847, Abcam, 1:1000), GFAP (16825-1-AP, Proteintech, 1:1000), NeuN (66836-1-Ig, Proteintech, 1:1000), p21^Cip1^ (SC6246, Santa Cruz, 1:1000), STAT3 (#9139, Cell Signaling Technology, 1:1000), p16^Ink4a^ (SC1661, Santa Cruz, 1:1000), p53 (#2524, Cell Signaling Technology, 1:1000), STAT3^Tyr705^ (#9145, Cell Signaling Technology, 1:1000), STAT3^Ser727^ (#94994, Cell Signaling Technology, 1:1000), APP (#76600, Cell Signaling Technology, 1:1000), BACE1 (#5606, Cell Signaling Technology, 1:1000), β-Actin (#4967, Cell Signaling Technology, 1:1000) and Hsp90 (SC13119, Santa Cruz, 1:1000) at 4°C overnight. After incubation with horseradish peroxidase (HRP)-conjugated secondary antibodies (Beyotime, A0208/A0216, 1:2000), the membranes were visualized using an enhanced chemiluminescence kit (Millipore, WBKLS0500). Protein band density was quantified by ImageJ software.

#### Real-time PCR (RT-PCR)

Total RNA was extracted from whole brain cortex tissues or cells using TRIzol (Invitrogen, 15596018). cDNA synthesis was performed using the PrimeScript™ RT Reagent Kit with gDNA Eraser (TaKaRa, RR047B) for RT-PCR. Quantitative real-time PCR was conducted with SYBR Green Master Mix (Yeasen, 11202ES08). Gene expression levels were determined using the delta cycle threshold (Ct) method and normalized to the geometric mean of three stably expressed housekeeping genes: β-actin, 18S rRNA, and hypoxanthine phosphoribosyltransferase (Hprt). The fold change in AP-treated cells was calculated relative to the vehicle-treated control. The primer sequences are listed in Table S1.

#### Flow cytometry

BV2 cells, when reaching 80% confluence, were treated with IgG and AP at final concentrations of 1.2 mg/mL and 5 μmol/L, respectively, and incubated for 24 hours. Subsequently, fluorescent microspheres were added at a 1:800 dilution, and the cells were incubated for an additional 4 hours in a cell culture incubator. The cells were then collected into EP tubes, centrifuged at 400 g for 5 minutes, and the supernatant was discarded. The cells were washed once with PBS, followed by another centrifugation to remove the supernatant. Finally, the cells were resuspended in 0.3 mL of PBS containing 2% FBS and analyzed using a flow cytometer (BD LSR Fortessa X-20). Data were analyzed using FlowJo v10 software.

### Quantification and statistical analysis

All statistical analyses were performed using GraphPad Prism v9.0. Data are presented as the mean ± standard error of the mean (SEM); N = mice, n = images/sections of mouse. For IHC and IF analysis, three or more images from each group were randomly selected and analyzed using ImageJ. For two-group comparisons, an unpaired Student’s *t*-test was employed. For comparisons between multiple groups, one-way analysis of variance (ANOVA) followed by Bonferroni post hoc test was used. A P-value less than 0.05 was considered statistically significant. Statistical significance is indicated as ∗P < 0.05, ∗∗P < 0.01, ∗∗∗P < 0.001.
