# Supplementary material for: Andrographolide attenuates microglial senescence in Alzheimer’s disease mice by suppressing the STAT3 signaling
Source: iScience. 2026 May 20;29(6):116033. doi: 10.1016/j.isci.2026.116033 (PMC13214295; doi:10.1016/j.isci.2026.116033)
Supplement: Document S1. Figures S1–S6 and Table S1 [file mmc1.pdf]

iScience, Volume *29*

## Supplemental information

# Andrographolide attenuates microglial senescence in Alzheimer's disease mice by suppressing the STAT3 signaling

**Haochang Song, Meng Yang, Shengquan Wu, Qihui Dai, Weihong Qin, Weiheng Xie, Yuzhi Chen, Xiaoyun Jiang, Xiaojun Zhang, Xiuqin Deng, Chuang Ouyang, Yunman Zhang, Xinguang Liu, Yingjie Zhu, and Gonghua Huang**

**Figure.S1**

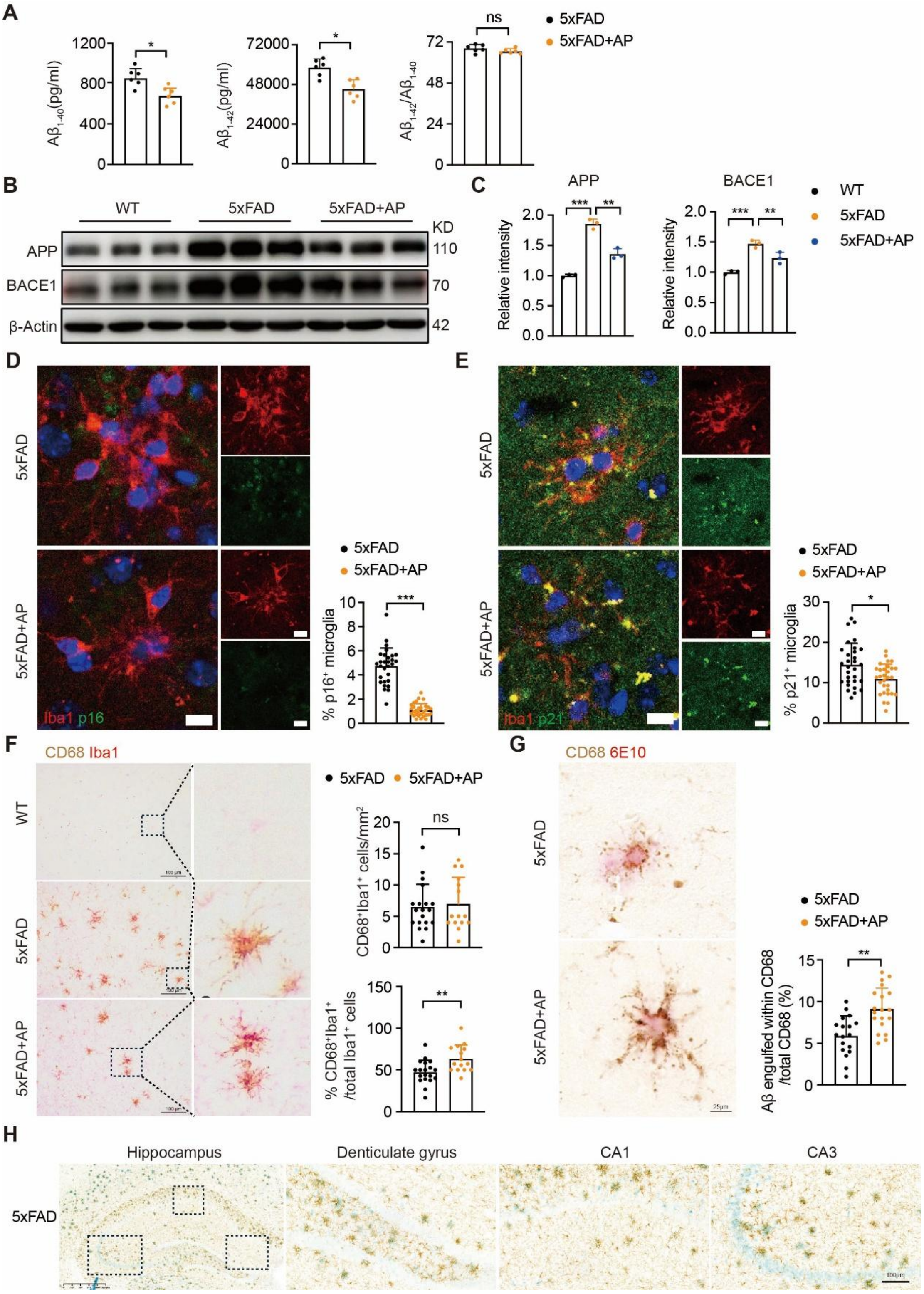
A
ns
5xFAD
5xFAD+AP
B
WT
5xFAD
5xFAD+AP
KD
APP
110
BACE1
70
β-Actin
42
C
APP
BACE1
Relative intensity
WT
5xFAD
5xFAD+AP
D
Iba1 p16
% p16⁺ microglia
E
Iba1 p21
% p21⁺ microglia
F
CD68 Iba1
WT
CD68⁺Iba1⁺ cells/mm²
% CD68⁺Iba1⁺ /total Iba1⁺ cells
G
CD68 6E10
Aβ engulfed within CD68 /total CD68 (%)
H
Hippocampus
Denticulate gyrus
CA1
CA3

**Fig. S1 AP reduced the level of A$\beta_{42}$, inhibits the expression of p16 and p21 proteins in 5×FAD mice, and enhances their phagocytic capacity**

(A) A$\beta_{1\text{-}40}$ and A$\beta_{1\text{-}42}$ measured by ELISA (N=5).

(B) Western blot analysis of APP and BACE1 expression in the cortex (N=3). Each data point represents the test result of one mouse.

(C) Quantitative data of APP and BACE1 protein levels (N=3). Each data point represents the test result of one mouse.

(D) Immunofluorescence staining with anti-Iba1 and anti-p16 antibodies, as well as quantitative statistics, in the cortex. Scale bar = 10 μm (5×FAD: N=5, n=5-6; 5×FAD+AP: N=5, n=6). Each data point represents the test result of one field of view.

(E) Immunofluorescence staining with anti-Iba1 and anti-p21 antibodies, as well as quantitative statistics, in the cortex. Scale bar = 10 μm (N=5, n=6). Each data point represents the test result of one field of view.

(F) Immunohistochemistry staining with anti-CD68 and anti-p16 antibodies, as well as quantitative statistics, in the cortex. Scale bar = 100 μm (5×FAD: N=5, n=3-4; 5×FAD+AP: N=5, n=3). Each data point represents the test result of one tissue slice. Sections were collected at approximately Bregma -1.70 mm (or immediately adjacent levels).

(G) Immunohistochemistry staining with anti-CD68 and anti-6E10 antibodies, as well as quantitative statistics, in the cortex. Scale bar = 25 μm (5×FAD: N=5, n=3-4; 5×FAD+AP: N=5, n=3-4). Each data point represents the test result of one tissue slice. Sections were collected at approximately Bregma -1.70 mm (or immediately adjacent levels).

(H) SA-β-gal staining of the hippocampal region in 5×FAD mouse brain. Scale bar = 625 or 100 μm. Data are expressed as mean ± SEM; N = the number of mice, n = number of fields of view per section from a single mouse. ANOVA followed by Bonferroni post hoc test, and unpaired Student's *t*-test were used. Statistical significance is indicated as: ns: not significant, *$P < 0.05$, **$P < 0.01$, ***$P < 0.001$.

## Figure.S2

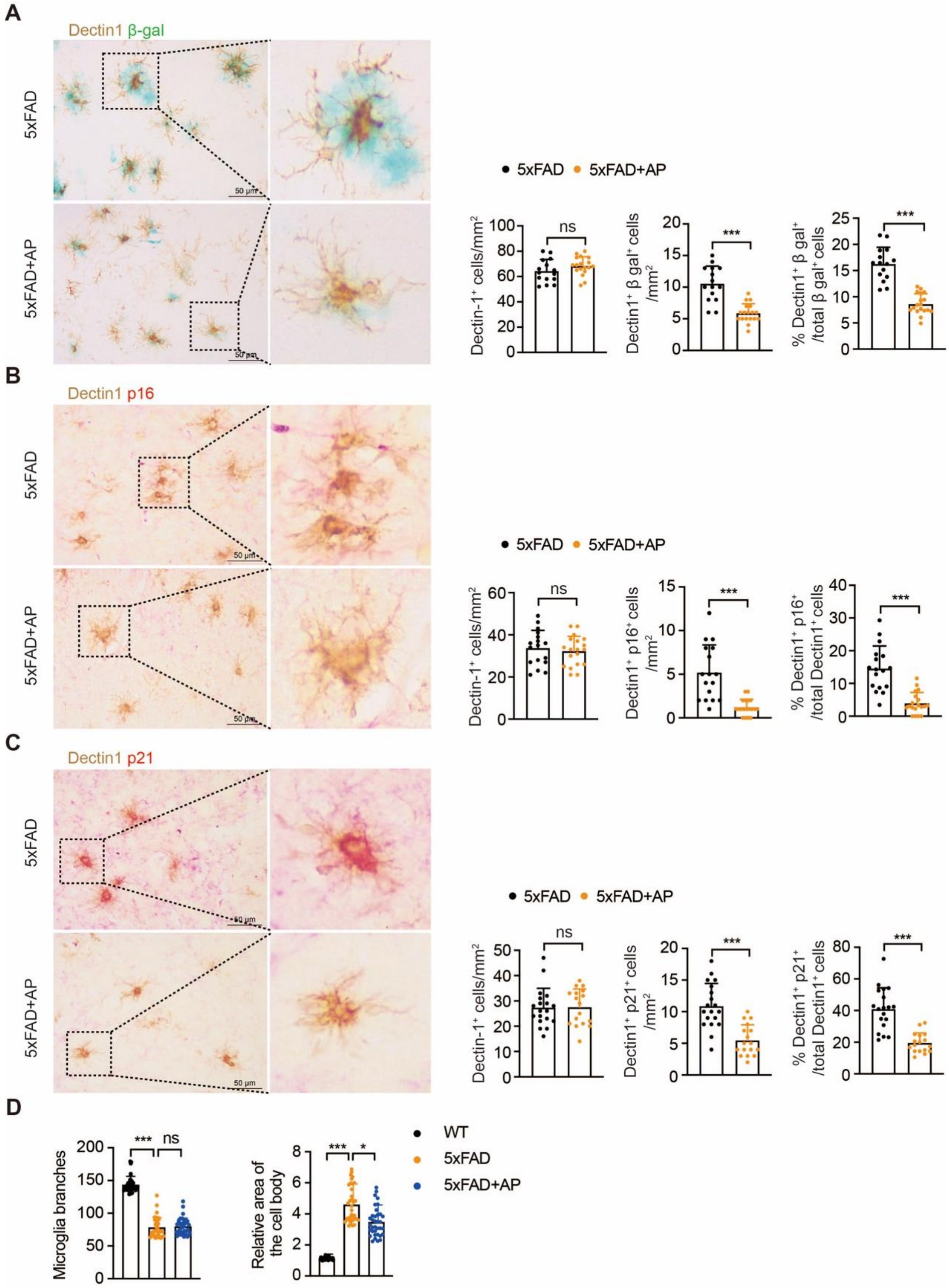

A
Dectin1 β-gal
5xFAD
5xFAD+AP
50 μm
5xFAD 5xFAD+AP
Dectin-1+ cells/mm2
Dectin1+ β gal+ cells /mm2
% Dectin1+ β gal+ /total β gal+ cells
ns
***
B
Dectin1 p16
Dectin-1+ cells/mm2
Dectin1+ p16+ cells /mm2
% Dectin1+ p16+ /total Dectin1+ cells
C
Dectin1 p21
Dectin1+ p21+ cells /mm2
% Dectin1+ p21+ /total Dectin1+ cells
D
Microglia branches
Relative area of the cell body
WT
5xFAD
5xFAD+AP
*

**Fig. S2 AP inhibits the senescence of disease-associated microglia and changes the morphology of microglia**

(A) Immunohistochemistry staining with anti-Dectin-1 antibody and β-gal staining, as well as quantitative statistics, in the cortex of 5×FAD mice. Scale bar = 50 μm (5×FAD: N=5, n=3; 5×FAD+AP: N=5, n=3-4). Each data point represents the test result of one tissue slice. Sections were collected at approximately Bregma -1.70 mm (or immediately adjacent levels).

(B) Immunohistochemistry staining with anti-Dectin-1 and anti-p16 antibodies, as well as quantitative statistics, in the cortex. Scale bar = 50 μm (5×FAD: N=5, n=3-4; 5×FAD+AP: N=5, n=3-4). Each data point represents the test result of one tissue slice. Sections were collected at approximately Bregma -1.70 mm (or immediately adjacent levels).

(C) Immunohistochemistry staining with anti-Dectin-1 and anti-p21 antibodies, as well as quantitative statistics, in the cortex. Scale bar = 50 μm (5×FAD: N=5, n=3-4; 5×FAD+AP: N=5, n=3-4). Each data point represents the test result of one tissue slice. Sections were collected at approximately Bregma -1.70 mm (or immediately adjacent levels).

(D) The number of microglial branches and the relative area of cell bodies (WT, 5×FAD and 5×FAD+AP: N=5, n=7). Each data point represents the test result of one cell. Data are expressed as mean ± SEM. N = the number of mice, n = number of fields of view per section from a single mouse. An unpaired Student's *t*-test was used. Statistical significance is indicated as ns: not significant, *P < 0.05, ***P < 0.001.

**Figure.S3**

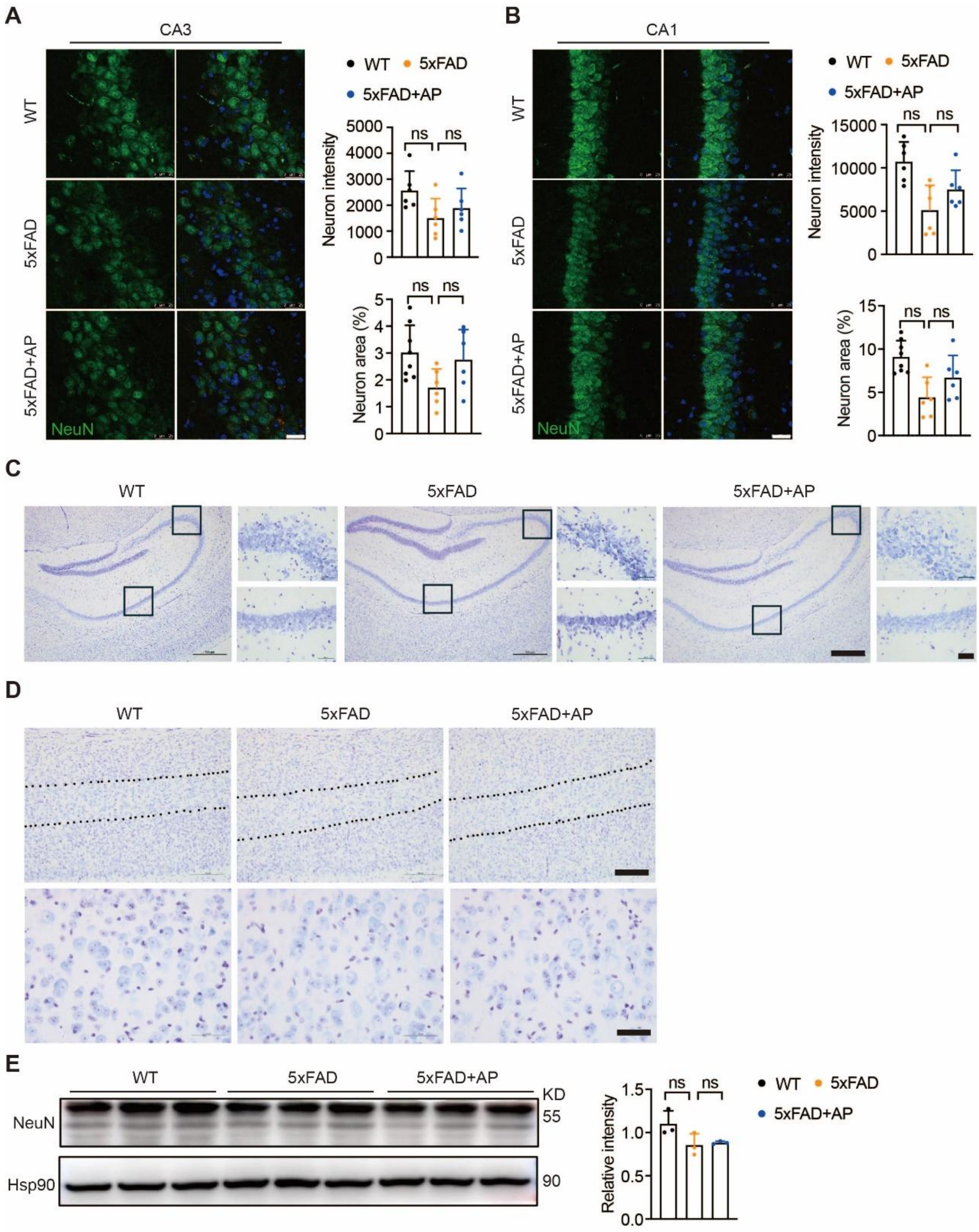
A
CA3
WT
5xFAD
5xFAD+AP
NeuN
WT 5xFAD
5xFAD+AP
Neuron intensity
5000
4000
3000
2000
1000
0
ns ns
Neuron area (%)
5
4
3
2
1
0
B
CA1
15000
10000
5000
0
15
10
5
C
D
E
KD
55
90
NeuN
Hsp90
Relative intensity
1.5
1.0
0.5
0.0

**Fig. S3 AP did not affect the density of neurons in the brain**

(A) Immunofluorescence staining of NeuN of the hippocampal CA3 region in 5×FAD mice and quantitative analysis of immunofluorescence. Scale bar = 25 μm (N=3, n=2). Each data point represents the test result of one tissue slice.

(B) Immunofluorescence staining of NeuN of the hippocampal CA1 region in 5×FAD mice and quantitative analysis of immunofluorescence. Scale bar = 25 μm (N=3, n=2). Each data point represents the test result of one tissue slice.

(C) Nissl staining of neuronal cells in the hippocampal CA3 and CA1 regions. Scale bar = 500 μm (large image) or 50 μm (small image) (N=3).

(D) Nissl staining of neuronal cells in the cerebral cortex. Scale bar = 200 μm (upper image) or 50 μm (lower image) (N=3).

(E) Western blot analysis of NeuN expression in the cortex and quantitative analysis (N=3). Each data point represents the test result of one mouse. Data are expressed as mean ± SEM. N = the number of mice, n = number of fields of view per section from a single mouse. ANOVA followed by Bonferroni post hoc test was used. Statistical significance is indicated as ns: not significant.

**Figure.S4**

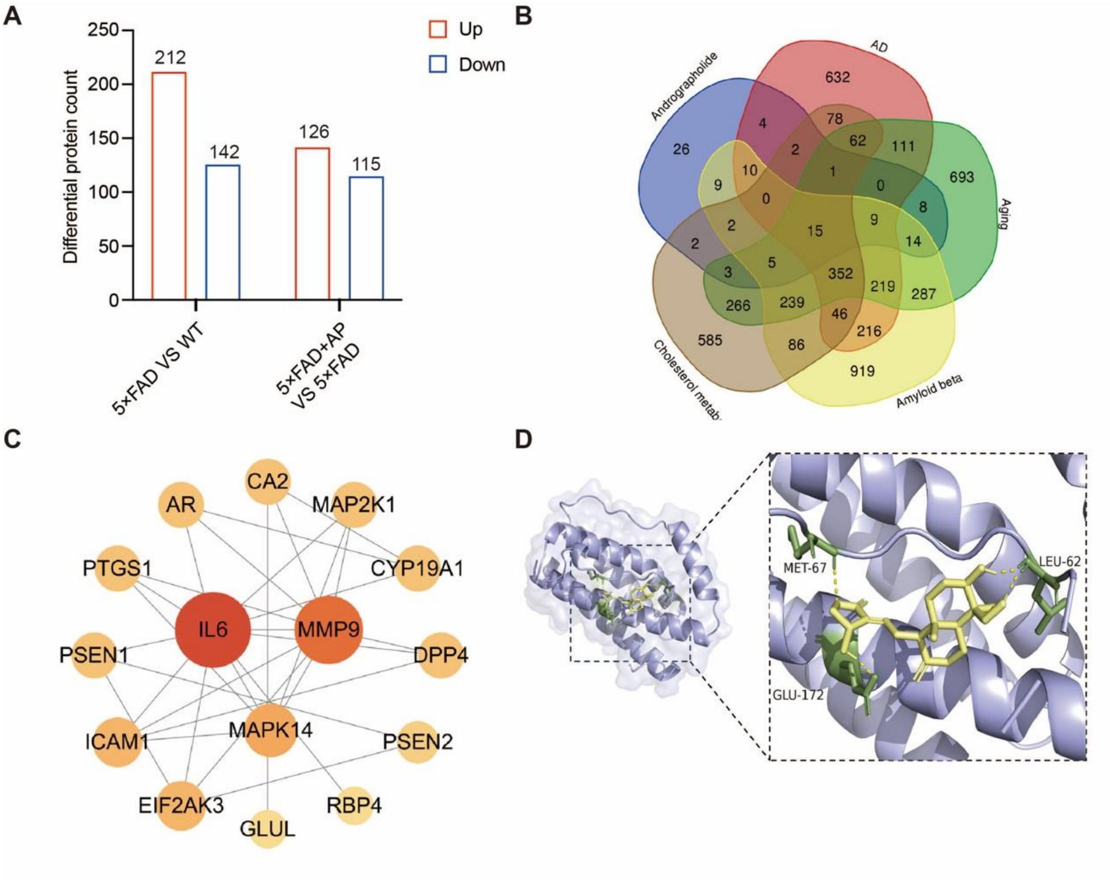
A
B
C
D
Up
Down
Differential protein count
250
200
150
100
50
0
212
142
126
115
5×FAD VS WT
5×FAD+AP VS 5×FAD
Andrographolide
AD
Aging
Amyloid beta
Cholesterol metab.
632
26
4
78
62
111
693
2
10
9
1
0
0
8
9
2
15
14
2
3
5
352
219
287
266
239
46
216
585
86
919
AR
CA2
MAP2K1
PTGS1
CYP19A1
IL6
MMP9
PSEN1
DPP4
ICAM1
MAPK14
PSEN2
EIF2AK3
GLUL
RBP4
MET-67
LEU-62
GLU-172

**Fig. S4 Screen for the common target genes of AP, Aβ, aging, AD and cholesterol metabolism**

(A) A histogram of all differentially expressed proteins in the cortex tissue.

(B) Venn diagram of the number of overlapping genes between AP, Aβ, aging, AD, and cholesterol metabolism.

(C) Protein-protein interaction network between AP, Aβ, aging, AD, and cholesterol metabolism. The edge between two nodes represents gene-gene interaction (unconnected nodes are hidden). The red inner circle indicates a degree value greater than 16.

(D) The molecular docking diagram of AP and IL-6 and the possible interacting residues.

**Figure.S5**

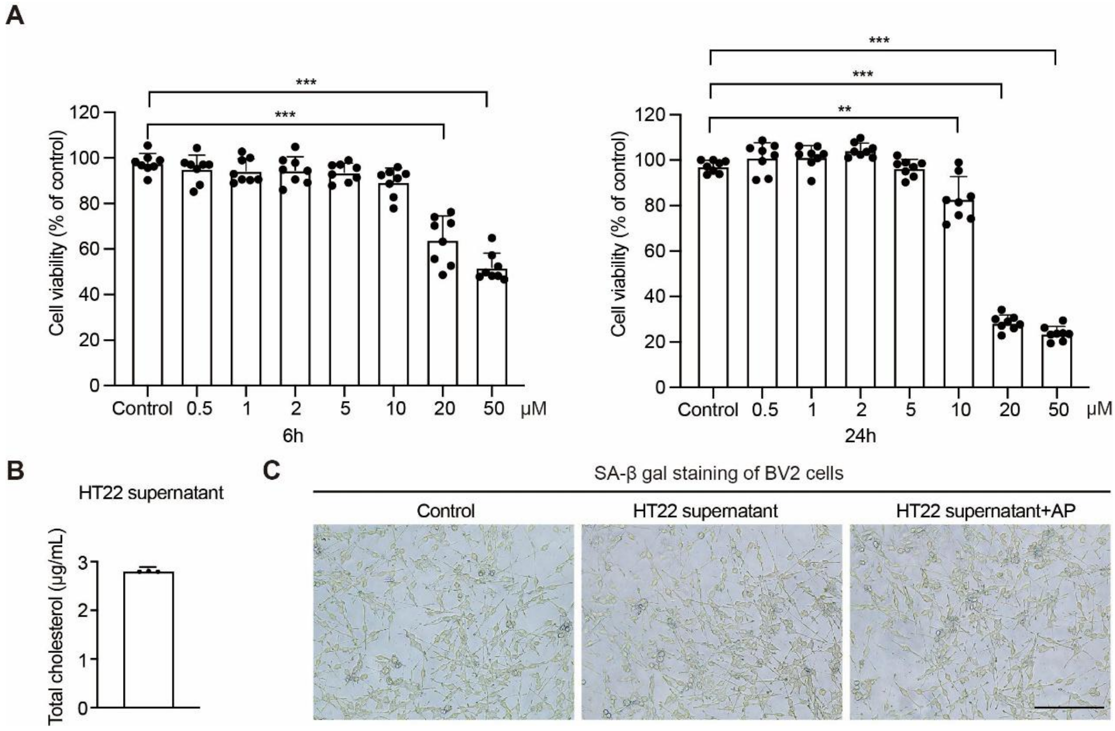
A
***
***
120
100
80
60
40
20
0
Cell viability (% of control)
Control
0.5
1
2
5
10
20
50
μM
6h
***
***
**
24h
B
HT22 supernatant
Total cholesterol (μg/mL)
3
2
1
0
C
SA-β gal staining of BV2 cells
Control
HT22 supernatant
HT22 supernatant+AP

**Fig. S5 Effect of AP on BV2 cell viability and influence of cholesterol on BV2 cell senescence**

(A) Effect of AP on BV2 cell viability. BV2 cells treated with AP for 6 hours (left panel) and 24 hours (right panel) (N=8). Each point represents a well.

(B) Cholesterol content in the supernatant of HT22 cells was measured by ELISA (N=3).

(C) SA-β-gal staining of coculture of microglia and neuron with or without AP treatment. Data are expressed as mean ± SEM. N = number of independent cell sample. An unpaired Student's *t*-test was used. Statistical significance is indicated as ns: not significant, $^{**}P < 0.01$, $^{***}P < 0.001$.

## Figure.S6

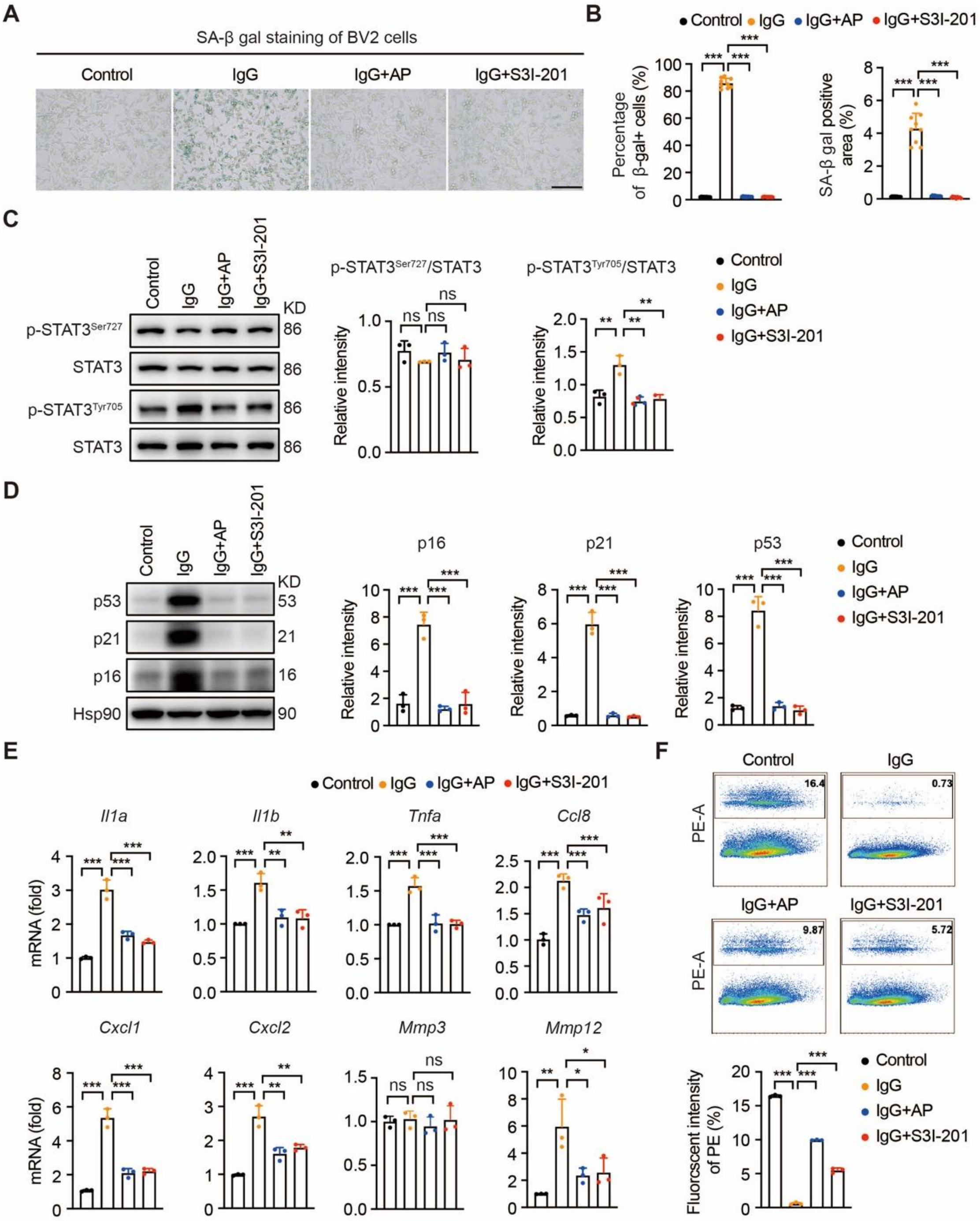
A
SA-β gal staining of BV2 cells
Control
IgG
IgG+AP
IgG+S3I-201
B
Control IgG IgG+AP IgG+S3I-201
Percentage of β-gal+ cells (%)
SA-β gal positive area (%)
C
p-STAT3Ser727
STAT3
p-STAT3Tyr705
KD
86
p-STAT3Ser727/STAT3
p-STAT3Tyr705/STAT3
Relative intensity
D
p53
p21
p16
Hsp90
53
21
16
90
E
Il1a
Il1b
Tnfa
Ccl8
Cxcl1
Cxcl2
Mmp3
Mmp12
mRNA (fold)
F
PE-A
16.4
0.73
9.87
5.72
Fluorscent intensity of PE (%)

**Fig. S6 AP inhibited IgG-induced STAT3 phosphorylation in BV2 cells**

(A) SA-β-gal staining of BV2 cells induced by IgG. Scale bar = 150 μm.

(B) Quantitative IgG-induced SA-β-gal staining (N=3, n=3). Each data point represents the test result of one field of view.

(C) Western blot analysis of STAT3 expression in IgG-induced BV2 cells and quantitative analysis (N=3). Each data point represents the test result of one sample and similar results were repeated in three biologically independent experiments.

(D) Western blot analysis of p16, p21 and p53 expression in IgG-induced BV2 cells and quantitative analysis (N=3). Each data point represents the test result of one sample and similar results were repeated in three biologically independent experiments.

(E) Quantitative PCR analysis of SASP factors in IgG-induced BV2 cells (N=3). Each data point represents the test result of one sample.

(F) Flow cytometry analysis of IgG-induced phagocytic function in BV2 cells and quantitative analysis of flow cytometry (N=3). Each data point represents the test result of one sample and similar results were repeated in three biologically independent experiments. Data are expressed as mean ± SEM. N = number of independent experiments, n = number of fields of view analyzed for each experiment. ANOVA followed by Bonferroni post hoc test was used. Statistical significance is indicated as ns: not significant, *P < 0.05, **P < 0.01, ***P < 0.001.

**Supplemental Table 1**

Primers for RT–qPCR.

| Gene | Sequence (5'-3) |
| --- | --- |
| *p53-F* | CCGACCTATCCTTACCATCATCA |
| *p53-R* | AGGCACAAACACGAACCTCAA |
| *Mmp3-F* | GTTGGA GAACATGGAGACTTTGT |
| *Mmp3-R* | CAAGTTCATGAG CAGCAACCA |
| *Mmp12-F* | TGCACTCTGCTG AAAGGAGTCT |
| *Mmp12-R* | GTCATTGGAATTCTGTCC TTTCCA |
| *Ccl8-F* | CGGGTGCTG AAAAGCTACGA |
| *Ccl8-R* | TTGGTCTGGAAAACCACAGCTT |
| *Cxcl1-F* | ACCGAAGTCATAGCCACACTC |
| *Cxcl1-R* | CTCCGTTACTTGGGGACACC |
| *Cxcl2-F* | CCAGACAGAAGTCATAGCCAC |
| *Cxcl2-R* | TGGTTCTTCCGTTGAGGGAC |
| *Tnfa-F* | ATGTCTCAGCCTCTTCTCATTC |
| *Tnfa-R* | GCTTGTCACTCGAATTTTGAGA |
| *$p16^{Ink4a}$-F* | CGCAGGTTCTTGGTCACTGT |
| *$p16^{Ink4a}$-R* | TGTTCACGAAAGCCAGAGCG |
| *Stat3-F* | TGTCAGATCACATGGGCTAAAT |
| *Stat3-R* | GGTCGATGATATTGTCTAGCCA |
| *$p21^{Cip}$-F* | CCTGGTGATGTCCGACCTG |
| *$p21^{Cip}$-R* | CCATGAGCGCATCGCAATC |
| *Lxra-F* | GAGAGGCTGCAACACACATA |
| *Lxra-R* | GAGGCTCACCAGCTTCATTAG |
| *Lxrb-F* | GCGTCCACCATTGAGATCAT |
| *Lxrb-R* | GTCCTTGCTGTAGGTGAAGTC |
| *Hmgcr-F* | AGCTTGCCCGAATTGTATGTG |
| *Hmgcr-R* | TCTGTTGTGAACCATGTGACTTC |
| *Hmgcs1-F* | AACTGGTGCAGAAATCTCTAGC |
| *Hmgcs1-R* | AACTGGTGCAGAAATCTCTAGC |
| *Sqle-F* | ATAAGAAATGCGGGGATGTCAC |
| *Sqle-R* | ATATCGAGAAGGCAGCGAAC |
| *Abca1-F* | AAAACCGCAGACATCCTTCAG |
| *Abca1-R* | CATACCGAAACTCGTTCACCC |
| *Il1a-F* | AGGGAGTCAACTCATTGGCG |
| *Il1a-R* | TGGCAGAACTGTAGTCTTCGT |
| *Il1b-F* | TTTGAAGTTGACGGACCCCAA |
| *Il1b-R* | CACAGCTTCTCCACAGCCACA |
| *Ldlr-F* | TGACTCAGACGAACAAGGCTG |
| *Ldlr-R* | ATCTAGGCAATCTCGGTCTCC |

Table 1 Continue

| Gene | Sequence (5’-3) |
|---|---|
| *Actin-F* | CTGAGAGGGAAATCGTGCGT |
| *Actin-R* | CCACAGGATTCCATACCCAAGA |
| *Hprt-F* | TCAGTCAACGGGGGACATAAA |
| *Hprt-R* | GGGGCTGTACTGCTTAACCAG |
| *18s-F* | TAAGTCCCTGCCCTTTGTACACA |
| *18s-R* | GATCCGAGGGCCTCACTAAAC |
